# Forced randomization: the what, why, and how

**DOI:** 10.1186/s12874-024-02340-0

**Published:** 2024-10-08

**Authors:** Kerstine Carter, Olga Kuznetsova, Volodymyr Anisimov, Johannes Krisam, Colin Scherer, Yevgen Ryeznik, Oleksandr Sverdlov

**Affiliations:** 1 Boehringer-Ingelheim Pharmaceuticals Inc, Ridgefield, CT USA; 2 Merck & Co., Inc., Rahway, NJ USA; 3 Amgen Ltd., London, UK; 4 Boehringer-Ingelheim Pharma GmbH & Co. KG, Ingelheim, Germany; 5 Rensselaer Polytechnic Institute, Troy, NY USA; 6 Department of Pharmacy, Uppsala University, Uppsala, Sweden; 7 Novartis Pharmaceuticals Corporation, East Hanover, NJ USA

**Keywords:** Drug supply chain management, Interactive response technology (IRT), Multi-center clinical trial, Poisson-gamma model

## Abstract

**Background:**

When running a randomized controlled trial (RCT), a clinical site may face a situation when an eligible trial participant is to be randomized to the treatment that is not available at the site. In this case, there are two options: not to enroll the participant, or, without disclosing to the site, allocate the participant to a treatment arm with drug available at the site using a built-in feature of the interactive response technology (IRT). In the latter case, one has employed a “forced randomization” (FR). There seems to be an industry-wide consensus that using FR can be acceptable in confirmatory trials provided there are “not too many” instances of forcing. A better understanding of statistical properties of FR is warranted.

**Methods:**

We described four different IRT configurations with or without FR and illustrated them using a simple example. We discussed potential merits of FR and outlined some relevant theoretical risks and risk mitigation strategies. We performed a search using Cortellis Regulatory Intelligence database (IDRAC) (www.cortellis.com) to understand the prevalence of FR in clinical trial practice. We also proposed a structured template for development and evaluation of randomization designs featuring FR and showcased an application of this template for a hypothetical multi-center 1:1 RCT under three experimental settings (“base case”, “slower recruitment”, and “faster recruitment”) to explore the effect of four different IRT configurations in combination with three different drug supply/re-supply strategies on some important operating characteristics of the trial. We also supplied the Julia code that can be used to reproduce our simulation results and generate additional results under user-specified experimental scenarios.

**Results:**

FR can eliminate refusals to randomize patients, which can cause frustration for patients and study site personnel, improve the study logistics, drug supply management, cost-efficiency, and recruitment time. Nevertheless, FR carries some potential risks that should be reviewed at the study planning stage and, ideally, prospectively addressed through risk mitigation planning. The Cortellis search identified only 9 submissions that have reported the use of FR; typically, the FR option was documented in IRT specifications. Our simulation evidence showed that under the considered realistic experimental settings, the percentage of FR is expected to be low. When FR with backfilling was used in combination with high re-supply strategy, the final treatment imbalance was negligibly small, the proportion of patients not randomized due to the lack of drug supply was close to zero, and the time to complete recruitment was shortened compared to the case when FR was not allowed. The drug overage was primarily determined by the intensity of the re-supply strategy and to a smaller extent by the presence or absence of the FR feature in IRT.

**Conclusion:**

FR with a carefully chosen drug supply/re-supply strategy can result in quantifiable improvements in the patients’ and site personnel experience, trial logistics and efficiency while preventing an undesirable refusal to randomize a patient and a consequential unblinding at the site. FR is a useful design feature of multi-center RCTs provided it is properly planned for and carefully implemented.

**Supplementary Information:**

The online version contains supplementary material available at 10.1186/s12874-024-02340-0.

## Introduction

Randomization is a well-established and widely accepted method of treatment assignment in a randomized controlled trial (RCT) [1]. Most RCTs enroll eligible participants sequentially, and for every subject in a sequence, the treatment group assignment is determined by chance, according to the randomization method chosen for the trial. Various randomization methods for RCTs are available [2]. Some recent papers discuss systematic approaches for choosing fit-for-purpose randomization methods for use in practice [3–5].

Many modern RCTs are run globally and utilize more than one research site (center) for study conduct. Multi-center RCTs enable broader coverage of patients across different geographies and can expedite the recruitment and completion of the study; however, they also pose some important methodological and practical challenges [6].

In practice, randomization is often implemented using the Interactive Response Technology (IRT). Two examples of IRT are the Interactive Web Response System (IWRS) and the Interactive Voice Response System (IVRS) [7]. IRT has many advantages for clinical trial management as it integrates tools for patient screening, recruitment, randomization, and monitoring, tools for drug supply management, and (sometimes) tools for electronic data capture [8]. In multi-center studies that use the IRT, one typically has two distinct randomization schedules: the subject randomization list and the medication pack list. The IRT enables central randomization, where subjects are randomized across the centers following one common randomization list. This is different from center-stratified randomization, where subjects within each center are randomized along their own center-specific randomization list. Central randomization might be stratified by several important prognostic factors in which case a separate randomization list is prepared for each stratum.

In a clinical trial with central randomization, an eligible participant is randomized to the treatment corresponding to the next available number in the randomization list, and then a pack of medication from the stock at the study center that has recruited the participant is dispensed. It is important to ensure scrambled kit IDs (for example, randomly permuted within the drug type) to prevent partial unblinding of the investigators through the divergence of the kit numbers [9]. Separating the randomization list and the medication pack list helps reduce the drug wastage, e.g., drug kits can be used interchangeably across subjects and multiple visits, and if a subject withdraws from the study, the unused drug may be allocated to other subjects in the same treatment group [10].

The drug supply at each study center should be sufficient to cover the patient demand. Since with central randomization treatment assignments at any center are practically independent, approaching complete randomization, the sequence of treatment assignments at any given center is completely unpredictable. Thus, ideally, all treatments should be available at any point of enrollment. However, in practice, this may be unrealistic due to various reasons. For example, study drug may be very expensive; the centers participating in the study may follow a competitive recruitment policy such that it is not known upfront how many patients will be recruited by a given center during a given study period; there could be large fast-recruiting centers where, due to chance, several patients might be randomized to the same treatment before drug re-supplies are received so that the site will run out of this type of drug; unforeseen disruptions of the drug supplies chain might occur in some regions, etc. The provisions for shortage of drug supply at study centers should be made at the trial planning stage.

The following options can be considered to address the stockout of some (but not all) types of medication at a study site. The first option is to have IRT issue a refusal to randomize the patient if the medication type for the treatment the patient was supposed to be randomized to is absent at the site. This option is problematic because it makes a patient, often a seriously sick person who made an effort to arrive at the site, to be refused randomization and treatment and return home, and possibly, discontinue the study because of this negative experience. It also causes a large frustration at the site who might pressure the sponsor for randomization to the available treatment at the site – or might keep the patient in the office trying to trick the system and call into IRT for randomization several times during the day in hopes that later in the day a treatment available at the site will come up for randomization. The awareness that one type of drug is absent at the site might also lead to partial or complete unblinding.

The second option is to oversupply the sites with drug to make sure the stockout never happens. This might require large initial drug stocks and lead to drug waste, especially when the randomization visit medication cannot be used for the later visits, or the study has several treatment arms. The third option, when the drug supplies are limited or expensive, is to use one of the dynamic allocation procedures designed for economical drug use, such as the modified Zelen’s approach [11, 12] or dynamic allocation with partial drug supplies sent to the study sites [13].

But often the most practical option is *forced randomization* (FR) [10] employed by IRT to randomize a subject when the corresponding treatment drug supply is not available at the site. With forced randomization, patients are allocated according to the pre-generated allocation schedule as long as the type of medication required for the randomization of the patient is available at their site. If such type of medication is not available at the site, the patient is allocated to the next free number in the randomization list that corresponds to a treatment that is available at the site [10]. The next patient to be randomized at a different site is allocated to the skipped treatment thus filling a gap in the allocation schedule (a technique called “backfilling”). Another approach is to cross out the skipped assignments from the randomization list and not use them for later randomized patients (“no backfilling” option). Thus, forced randomization can be considered an allocation schedule-guided procedure with a dynamic element that arises when the drug that corresponds to the next allocation is not available at the site. Forced randomization is mostly used in multi-center trials with central randomization not stratified by the study center because in the latter case the drug supply needs are fully predictable except in cases of the drug damage or dispensation error.

Forced randomization should not be confused with the replacement of a randomized subject who did not complete the study with another subject, as may be done in Phase I studies; it should also not be confused with restricted randomization where the allocation probabilities “force” a better balance in treatment arm totals at the end of randomization [2].

There are several advantages of designing an IRT to allow for FR. These advantages will be elaborated on momentarily. In essence, FR can help avoid refusals to randomize patients, which can cause frustration to the patients and the study personnel; it can also improve the trial logistics, drug supply management, and cost efficiency while maintaining the important statistical properties of the RCT. At the same time, FR does not eliminate the need for a careful drug supply planning – quite the opposite, the initial supplies and re-supply triggers should be set to ideally result in no FR, and if this is not feasible, result in an acceptably small fraction of forced allocations.^1^

There seems to be an industry-wide consensus that using FR can be acceptable in confirmatory trials provided there are “not too many” instances of forcing; however, there is no guidance on what number accounts for “too many” forced allocations. The population-based analyses, most commonly employed in clinical trials, do not depend on the type of implemented randomization [2], and thus properly executed forced randomization that does not give rise to unblinding or bias, does not impact the analyses results. Therefore, the main goal of keeping the number of forced allocations low is to minimize any potential for bias that might arise if the forced allocation is not executed properly (as will be shown in the examples below). Typically, with properly executed forced allocation, the reason for an occasional stockout is a random phenomenon of several allocations to the same treatment arm at the site that happen faster than the drug re-supply can arrive. For most studies, the spikes of enrollment of several patients randomized at the site within a day or two are rare [14]; having most of these subjects randomized to the same arm makes it even less likely. Small percentage of forced allocations, below or around 5% achievable with reasonable drug supply strategies as the simulations below demonstrate, will result in the properties of the allocation procedure similar to those of the original procedure (as seen in [15]). Thus, the goal of minimizing the potential for bias can be achieved by focusing on the diligent drug supply management that avoids massive stockouts or unacknowledged by the sites drug resupplies and ensures that the reason for drug stockout is the random occurrence of several allocations to the same treatment at the site in a time interval too short for the re-supply drugs to arrive.

A better understanding of statistical properties of FR is warranted and will be pursued by the authors in the follow-up work.

In this paper, we explore the phenomenon of FR in detail. In the next section, we describe different types of FR and examine its features through a simple example. In section “Potential merits and risks associated with forced randomization” we review some advantages of FR, list some relevant potential risks and outline risk mitigation strategies. In section “How frequent is the use of forced randomization option in practice?” we present the results of a search using Cortellis Regulatory Intelligence database (www.cortellis.com) to understand the reported use of FR in clinical trials. In section “Planning for the forced randomization option at the trial design stage” we propose and conduct a simulation study to assess the impact of FR for a hypothetical clinical trial. Section “Conclusion” provides a summary of our findings and discusses some future work.

## Defining forced randomization and examining its features: The “What”

**Fig. 1:**
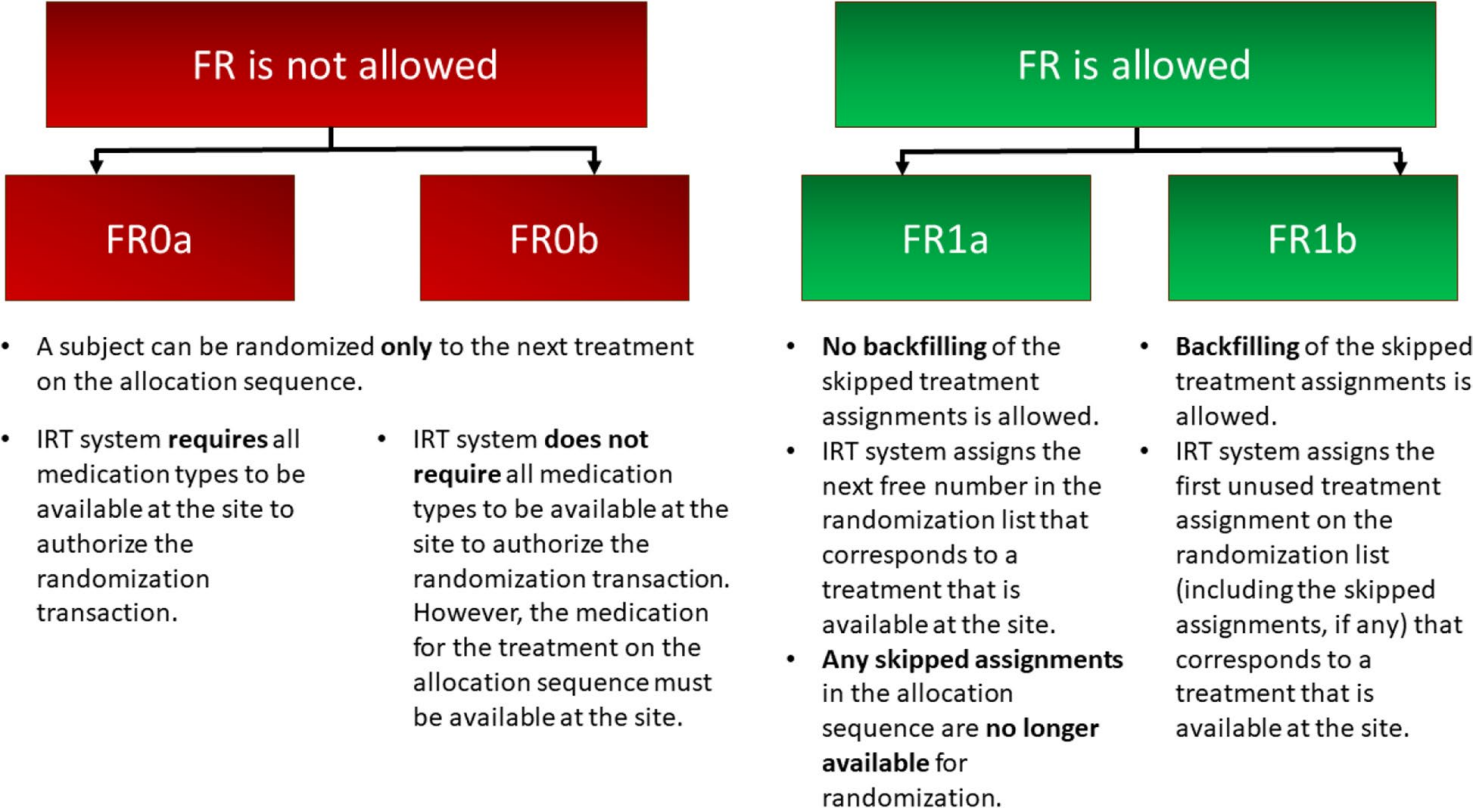
Four different configurations for randomization in an IRT

In this paper, we will use the term “forced randomization” in accordance with the definition provided in [10], whereby forced randomization may occur when a site runs out of one particular type of medication but still has stocks of other treatments remaining, in which case the randomization may be restricted to the treatments for which the stocks of medication are available at the site. In many modern IRT systems, one can have four different configurations to facilitate randomization in a multi-center study (Fig. 1).

FR0a: FR is not allowed, that is, a subject can be randomized only to the next treatment on the allocation sequence. Moreover, the IRT system does not authorize the randomization transaction unless all medication types are available at the site.

FR0b: FR is not allowed, but the IRT system does not require all medication types to be available at the site to authorize the randomization; i.e., only the medication for the next treatment on the allocation sequence needs to be available for randomization.

FR1: FR is allowed; the difference between the configurations FR1a and FR1b is in how the subjects are randomized after the forced randomization took place:

FR1a does not allow backfilling of the skipped assignments on the allocation list. The IRT system assigns the next free number in the randomization list that corresponds to a treatment that is available onsite. Any skipped assignments on the allocation sequence are no longer available for randomization.

FR1b allows backfilling of the skipped assignments. The IRT system assigns the first unused assignment on the randomization list (including previously skipped assignments, if any) that corresponds to a treatment that is available onsite.

**Fig. 2:**
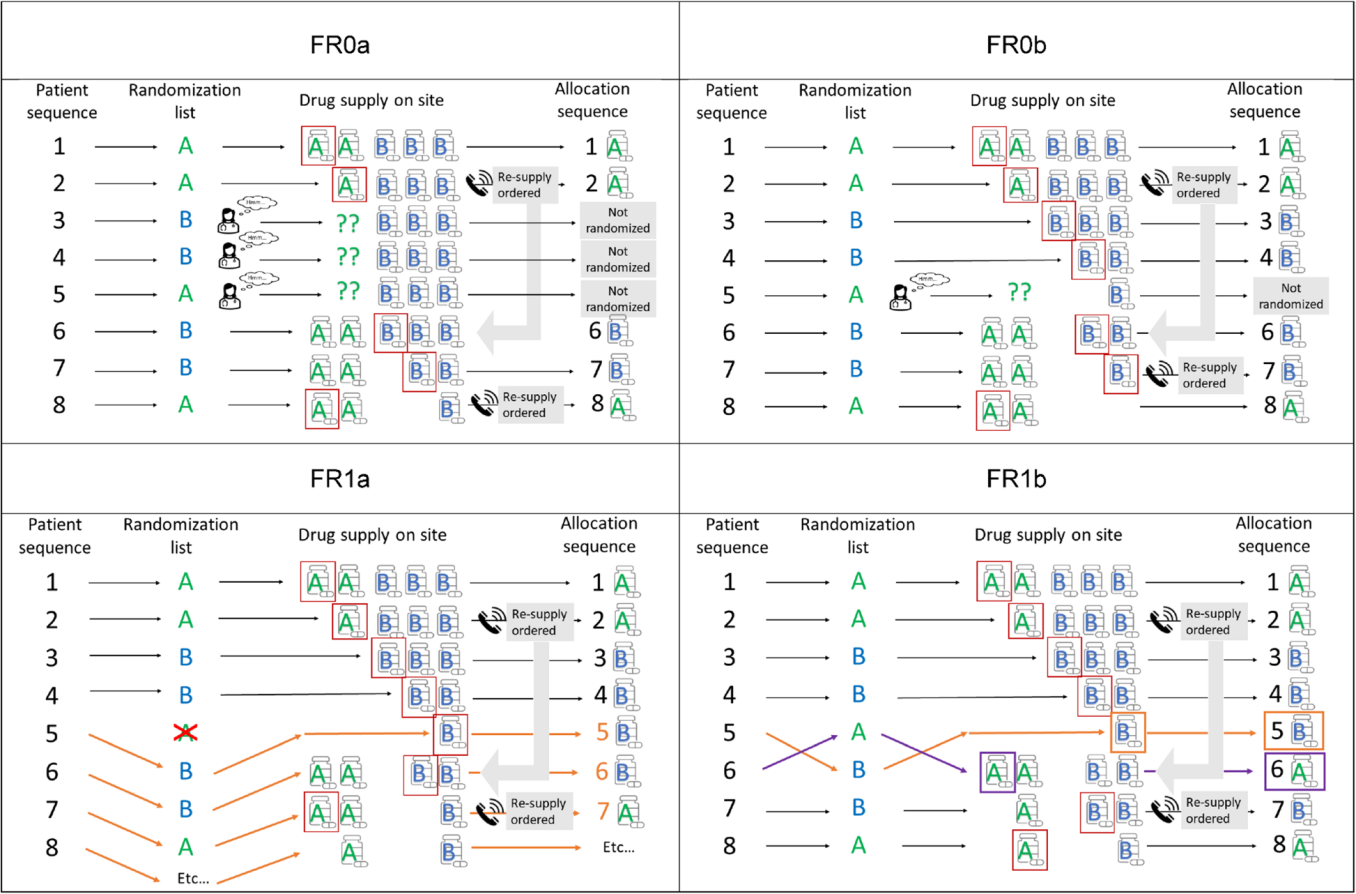
The randomization flow for a “toy” example. Description for Figure 2: We have a 1:1 RCT with the permuted blocks of 4 as the method of randomization. The randomization list for the first two blocks is |AABB|ABBA|. Initially, there are two kits of drug A and three kits of drug B onsite. Patients are enrolled sequentially and must be randomized to treatment immediately. For patients 1 and 2, the randomized treatment assignment will be made consistently with the randomization list with all four IRT configurations. Subsequently, the randomized assignments will depend on the IRT configuration. If the IRT system is configured as FR0a, then patients 3, 4, and 5 will be sent home without being randomized because no drug A is available on site (therefore, randomization transactions are not authorized). The drug has been re-supplied at patient 6’s entry, and patients 6, 7, and 8 are randomized to B, B, and A, as scheduled. If the IRT system is configured as FR0b, then patients 3 and 4 are randomized to drug B as scheduled because drug B is available on site, and randomization transactions are authorized. Patient 5 will be sent home without being randomized because of the lack of drug A supply on site. The drug has been re-supplied at patient 6’s entry, and patients 6, 7, and 8 are randomized to B, B, and A, as scheduled. If the IRT system is configured as FR1a, then patients 3 and 4 are randomized to drug B as scheduled, but patient 5 cannot be randomized to drug A because no drug A is available on site. Instead, patient 5 is “forced-randomized” to the next free number in the randomization list that corresponds to a treatment that is available (drug B in our example), and the fifth allocation (A) is crossed out to show that it can no longer be assigned. The drug has been re-supplied at patient 6’s entry, and patients 6, 7, and 8 are randomized to treatments that appear consecutively on the randomization list. If the IRT system is configured as FR1b, then patients 3 and 4 are randomized to drug B as scheduled, patient 5 is “forced-randomized” to receive drug B (because it is available), and the fifth allocation (A) will be provisioned for the next patient. The drug has been re-supplied at patient 6’s entry, and patient 6 is “forced-randomized” to drug A to backfill the previously unused position in the randomization list. Patients 6 and 7 are randomized to B and A, as scheduled

To appreciate the difference between the IRT configurations, consider a randomized, parallel group, equal allocation trial comparing the effects of two treatments, A and B. For simplicity of illustration, let us assume that the study has a single center (although in practice FR is mostly used in multi-center trials with central randomization not stratified by study center). Suppose the randomization is performed using permuted blocks of length 4 such that for every four participants there are exactly 2 allocations to each of the treatments A and B. Suppose the randomization list for the first two blocks is |AABB|ABBA| and assume that initially there are two kits of drug A and three kits of drug B at the site (Fig. 2).

Assume that the first three patients are recruited by the site on the same day. Patients #1 and #2 are enrolled and both are assigned to treatment A according to the randomization list and based on the availability of drug A at the site. Note that after the first two patients have been randomized, there is a shortage of drug A at the site, and so a re-supply order will be made (Fig. 2, Call icon). It takes some time for the drug to be shipped and delivered, and in our example, we assume the shipment will be available at the site only by the time when patient #6 is enrolled.

When patient #3 is enrolled, only drug B supplies are available. If the IRT system is configured as FR0a (Fig. 1, Option FR0a), then the system first performs a check to see if all medication kit types (i.e., both A and B) are available, and only if this is the case, can a randomization transaction be authorized. In our example, this is not the case because drug A is not available, and thus patient #3 will be sent home without being randomized (some consequences of such a decision shall be discussed momentarily). On the other hand, if the IRT system is configured as FR0b (Fig. 1, Option FR0b), then the IRT system skips the check of all medication kit types and performs the treatment assignment for the patient according to the randomization list only if that medication is available. In our example, this is indeed the case (i.e., the randomized assignment for patient #3 is B, and drug B is available); therefore, with FR0b patient #3 will be randomized to treatment B as intended.

For patient #4, the intended randomized assignment is treatment B, which is available at the site. However, with FR0a configuration, the randomization transaction will not be authorized due to the lack of drug A supply, and, therefore, patient #4 will be sent home without being randomized. By contrast, with three other configurations (FR0b, FR1a, and FR1b), patient #4 will be enrolled and randomized to treatment B as intended.

Let us consider the time point when patient #5 is enrolled. With both FR0a and FR0b, patient #5 will be sent home without being randomized because of the lack of treatment A at the site. However, with FR1a (Fig. 1, Option FR1a) and FR1b (Fig. 1, Option FR1b), patient #5 will be randomized to the next free number in the randomization list that corresponds to a treatment that is available (treatment B in our example). In other words, patient #5 will “skip” the fifth position of treatment A in the randomization list and will be “forced allocated” to treatment B that appears in the sixth position in the randomization list (|AABB|A**B**BA|). Note that the fifth position will be left unused at that time.

When patient #6 is enrolled, the medication re-supply has taken place, and both treatments A and B are available at the site. With both FR0a and FR0b, patient #6 will be randomized to treatment B according to the randomization list. With FR1a, the IRT system is configured to not allow backfilling of the skipped assignments, and so patient #6 will be assigned to the next free number in the randomization list that corresponds to a treatment available at the site (treatment B in the seventh position in the randomization list (|AABB|B**B**A|; treatment A in the 5th position is crossed out to show that it can no longer be assigned). With FR1b, patient #6 will “backfill” the skipped treatment A in the 5th position in the randomization list (|AABB|**A**BBA|).

Finally, when patients #7 and #8 are enrolled, the randomized treatments are available, and so these patients will be randomized as scheduled (with FR1a, the new block in the randomization list will be utilized).

## Potential merits and risks associated with forced randomization: The “Why”

### Merits

FR can improve patient burden as well as the flexibility of the trial logistics in several ways. First, the FR option avoids sending an eligible patient back home and depriving him or her from the opportunity to participate in the clinical trial – an ethical issue. Losing eligible patients can be extremely frustrating to the site that knows that some drug supplies are available, and it can also damage the sponsor relationship with the site. This could be especially damaging in studies of rare disorders where eligible participants are very hard to find. Some sites might also resort to inappropriate ways of dealing with such situation, e.g., giving the subject a drug kit available at the site without IRT randomization, or (with configuration FR1) waiting for a few hours and trying randomization again in hope that some patients are randomized at other sites and the drug available at the site will now be assigned. Forced randomization, on the contrary, guarantees an ethical treatment of the patient and the site personnel.

Second, a refusal to randomize a patient unblinds the site to the fact that at least one drug type is absent at the site which might lead to partial unblinding. In a 2-arm study, if several kits are present at the site when a refusal to randomize happened, the site knows these kits are of the same type and can later track subjects assigned to the same treatment, which increases the potential for unblinding through observed outcomes. In contrast, having an FR option built in the IRT system allows for continuing the enrollment without a site being aware of the lack of a drug type at the site and thus removing the possibility of unblinding.

Third, the FR option allows to reduce the drug stocks at the site, which is especially impactful when the drug is novel and not available in large quantities. Thus, FR has a potential to accelerate the new drug development and bring the novel therapies to the patients faster. It can also reduce the drug costs when the drug is expensive and thus enable the clinical research. Overall, the need for FR is higher in trials where the drug is administered only once and thus the sites do not have additional supplies for later visits that can be used for the randomization visit as well. Studies with multiple treatment arms, more common nowadays, might also have an increased need of an FA option.

Fourth, if properly implemented, the forced randomization with the backfilling option can promote a good balance in treatment assignments as most gaps in the allocation sequence caused by forced allocations are filled later.

Finally, including an FR option provides some mitigation to unforeseen temporary disruptions in the drug distribution logistics caused by natural disasters, pandemics, or other large-scale problems [16].

### Limitations, risks, and risk mitigation strategies

In some circumstances, FR may lead to undesirable consequences such as partial unblinding and potential for bias. The awareness of such possibility helps ensuring that the proper steps that prevent the unblinding and bias are taken. Table 1 provides some examples (albeit the list is not exhaustive) of potential risks associated with FR, types of bias that can arise, and possible risk mitigation strategies. Note that the mentioned risks should be regarded as theoretical concerns that could potentially arise under certain circumstances. In practice, normally neither the investigators nor the trial statisticians would be looking for clues that could be provided by FR. However, it may be prudent to review and assess the potential for such risks at the study planning stage and specify the parameters of the IRT accordingly.

**Table 1:** Theoretical risks associated with forced randomization and the corresponding risk mitigation strategies

Phenomenon	Example	Risks	Risk mitigation
Temporary shortage of supply of a certain drug type	Suppose an experimental drug (E) is not available at any of the sites until the new batch is produced, but the study continues enrolling participants. If FR is allowed = > skipped allocations are known to belong to treatment E. When drug is re-supplied, massive backfillings to the skipped allocation numbers will be occurring.	Observer bias Selection bias Chronological bias	Option 1. Design the study to use scrambled allocation numbers to conceal that forced allocation occurred; this prevents the selection bias and the observer bias, but not the chronological bias associated with the time trend. Option2. If feasible, continue screening the participants but pause the enrollment until drug supplies become available on site.
Potential unblinding in a study with interchangeable use of placebo run-in and active placebo kits	Suppose we have a two-period 1:1 RCT, where both arms have placebo run-in Period 1 followed by a randomized (Active: Placebo) Period 2 = > plenty of placebo supplies at the sites during randomization. If FR is allowed = > skipped allocations belong to Active and actual assignments are Placebos.	Selection bias	Use scrambled allocation numbers to prevent the knowledge of whether the participant was assigned according to the original randomization or using FR resolves the issue.
Potential unblinding through FR in a study with a highly unequal allocation ratio	Suppose the initial drug supply to each site includes one block of kits (say, 5 + 1 drug kits in a study with 5:1 randomization, A:B). If it is known that some allocation has been skipped before 5 patients are randomized and before re-supply arrives at the site = > skipped allocation is known to be B.	Observer bias	Use scrambled allocation number to conceal occurrences of FR. Also, include more than one kit of drugs of the rare treatment group both in the initial and in the re-supply shipments
Improper specification of the re-supply triggers	Suppose the study has unequal allocation ratio (e.g., 3:1, Active: Placebo) but uses equal re-supply triggers (e.g., request re-supply kits of a certain type if < 3 kits of this type are available). If at some point new shipments are delayed but the site continues randomizing new patients until the supplies are exhausted = > greater than desired allocation to Placebo.	Unblinding Selection bias	Carefully specify the drug resupply policy. Use scrambled allocation numbers to prevent the knowledge of whether the participant was assigned according to the original randomization or using FR.
Site forgets to acknowledge the receipt of resupplies	IRT, not having an accurate picture of what is available at the site, force-allocates several subjects at the site to the same treatment.	Lack of within-center balance; possible bias	Ensure strong compliance with the drug receipt acknowledgement processes.

FR may be at odds with the ICH E9 guidance [1] which specifies:

The next subject to be randomized into a trial should always receive the treatment corresponding to the next free number in the appropriate randomization schedule (in the respective stratum, if randomization is stratified).

For the studies with FR, we suggest the following modification of the rule above:

The next subject to be randomized into a trial receives the treatment corresponding to the first free number (that corresponds to the available kits at the site) in the appropriate randomization schedule (in the respective stratum, if randomization is stratified).

In studies with backfilling, the first free (unused) number on the schedule might be followed by one or more numbers already assigned to force-randomized subjects. In studies without backfilling, when a patient is force-randomized, all numbers preceding his or her allocation on the allocation schedule are made unavailable (no longer free to be assigned). Such unfilled gaps in the allocation schedule might lower the balance in treatment assignments at the end of randomization.

While the ICH E9 guidance was developed before the wide adoption of IRT, since than the broad use of IRT in multi-center clinical trials made central randomization, where subjects are randomized across the centers rather than according to a center-stratified schedule, an easily available and most commonly used option. The need to support often unpredictable drug needs at the sites leads to the FR option [10].

Since no regulatory guidance on the topic of FR is available in publications, a common question that arises at study design is “How many forced allocations are acceptable in the study?” Setting a limit (a cap) on the number of forced allocations does not solve the problem of how to handle the unexpected drug shortages: it just pushes the problems faced in absence of required drug later in the enrollment. Instead, a careful drug supply planning and execution is required to minimize the percentage of forced allocations.

Overall, the need for careful specification of all details of FR (e.g., a mention of that only the drug supply manager is notified of instances of forced allocation, the use of scrambled allocation numbers, etc.) cannot be underestimated. When sequential patient IDs (allocation numbers on the randomization schedule) are used in a study, a person with access to the complete sequence of allocation numbers and dates/times of randomization might be able to identify instances of forced allocation by looking at allocation numbers deviating from the chronological order. In practice, only the dates of randomization (not times) are typically available in the data collection systems; moreover, these dates are collected locally, according to the time zone of the site. Thus, the exact order of allocation typically cannot be derived from available data, and thus only some, but not all forced allocations can be identified other than by the IRT system. Nevertheless, if potential for partial unblinding caused by identified forced allocations is a concern, the IRT can be set up to use scrambled allocation numbers. Using scrambled allocation numbers is generally a good practice.

## How frequent is the use of forced randomization option in practice?

**Fig. 3:**
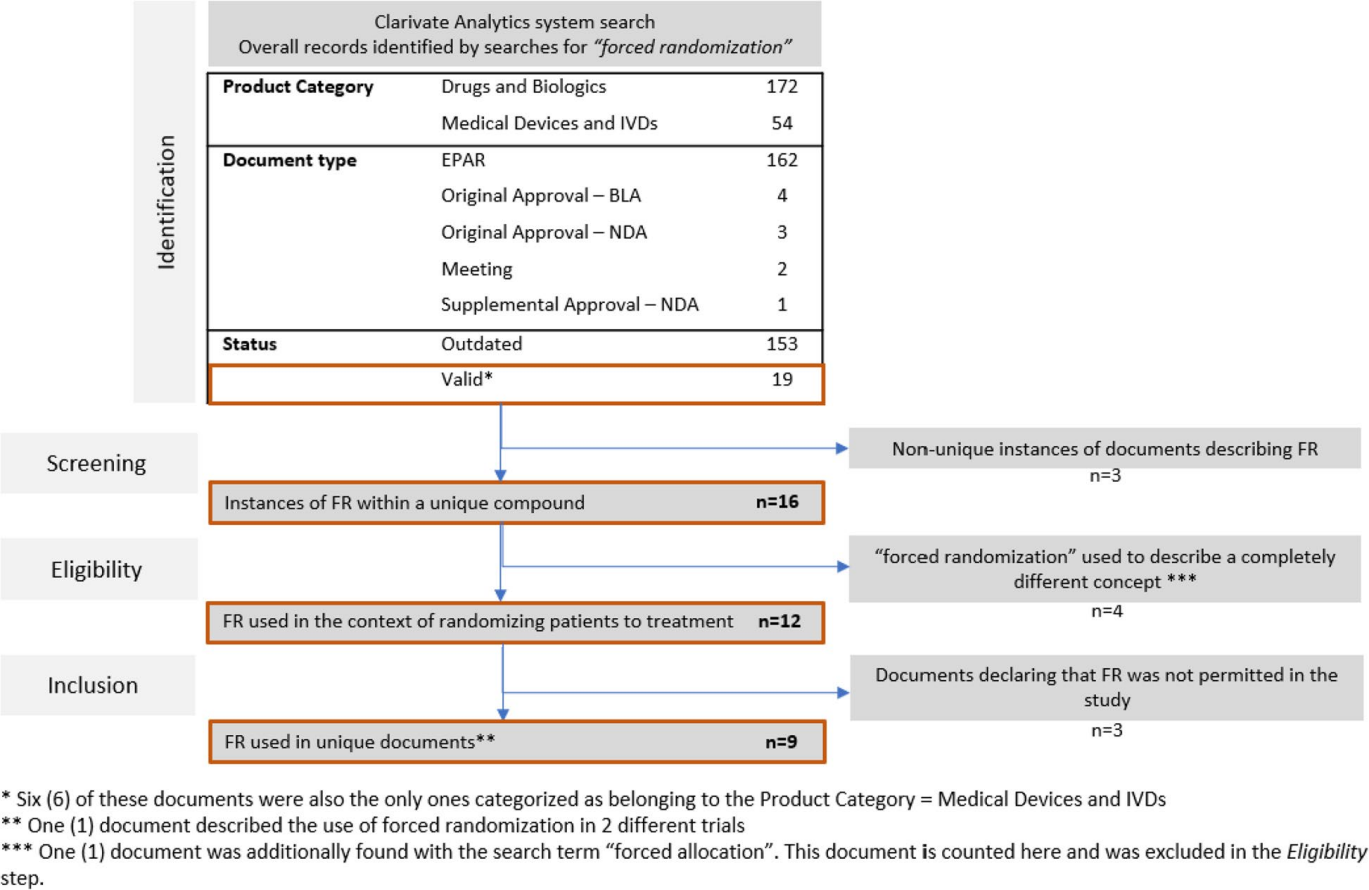
Document selection process in Cortellis using the search term = “*forced randomization*”. Abbreviations: IVD = In vitro diagnostics; EPAR = European public assessment report; BLA = Biologic License Application; NDA = New Drug Application

To gain a better understanding of the prevalence of FR in clinical trial practice, we conducted a search using the Cortellis Regulatory Intelligence database (IDRAC) (www.cortellis.com). Figure 3 summarizes the document selection process.

We specified the search term “*forced randomization*” among the documents of the Report type = *Regulatory*. As of October 10, 2023, a total of 19 documents with Status = *Valid* were identified. After further scrutiny of the identified documents (cf. Fig. 3), a total of 9 documents were included in the analysis. The characteristics of these documents are summarized in Table 2.

**Table 2:** The characteristics of the studies identified in Cortellis (by IDRAC number) that mentioned the use of forced randomization (FR) A total of 9 submission documents were found. One submission (IDRAC number 234486) included two studies, MODIFY I (ClinicalTrials.gov: NCT01513239) and MODIFY II (ClinicalTrials.gov: NCT01241552), and mentioned the use of FR without providing further details on the actual number of FRs in these studies. Another submission (IDRAC number 361985) was based on the pooled data from two studies, RE-COVER (ClinicalTrials.gov: NCT00291330) and RE-COVER II (ClinicalTrials.gov: NCT00680186); therefore, the reported values (sample size, number of FRs, etc.) are based on the pooled data from these two studies

IDRAC Number	Study name [main publication reference]	ClinicialTrials.gov identifier	Country/ Region	Document Type	Sample size	Allocation ratio	Stratification	Block size	Number of FRs	% FR^a^
363,676	ECOSPOR III - SER-109 Versus Placebo in the Treatment of Adults With Recurrent Clostridium Difficile Infection (ECOSPORIII) [17]	NCT03183128	USA	Original Approval - BLA	182	1:1	2 strata, 2 levels	unavailable	6	3.30%
304,185	Study of a Novel Antipsychotic ITI-007 in Schizophrenia [18]	NCT01499563	USA	Original Approval - NDA	335	1:1:1:1	none	unavailable	11^b^	3.28%
308,579	CAROLINA: Cardiovascular Outcome Study of Linagliptin Versus Glimepiride in Patients With Type 2 Diabetes [19]	NCT01243424	USA	Supplemental Approval - NDA	6033	1:1	by center	unavailable	98	1.62%
137,950	Study Comparing 13-valent Pneumococcal Conjugate Vaccine With 7-valent Pneumococcal Conjugate Vaccine [20]	NCT00373958	USA	Meeting	666	1:1	by center	unavailable	3	0.45%
234,486	A Study of MK-6072 and MK-3415 A in Participants Receiving Antibiotic Therapy for Clostridium Difficile Infection (MK-3415 A-002) (MODIFY II) [21]	NCT01513239	USA	Original Approval - BLA	1168	1:1:1	2 strata, 4 levels	unavailable	Not reported	--
	A Study of MK-3415, MK-6072, and MK-3415 A in Participants Receiving Antibiotic Therapy for Clostridium Difficile Infection (MK-3415 A-001) (MODIFY I) [21]	NCT01241552			1412	1:1:1:1	2 strata, 4 levels	unavailable	Not reported	--
351,428	A 12-Month Phase 3 Study of Pasireotide in Cushing’s Disease [22]	NCT00434148	European Union	EPAR	165	1:1	1 stratum, 2 levels	unavailable	1	0.61%
362,467	Placebo-Controlled Randomized Trial of Adjuvant Imatinib Mesylate Following the Resection of Localized, Primary Gastrointestinal Stromal Tumor (GIST) [23]	NCT00041197	European Union	EPAR	773	1:1	1 stratum, 3 levels	unavailable	60	7.76%
361,985	Efficacy and Safety of Dabigatran Compared to Warfarin for 6 Month Treatment of Acute Symptomatic Venous Thromboembolism (RE-COVER I) [24]	NCT00291330	European Union	EPAR	5331	1:1	2 stata, 4 levels	4	18	0.34%
	Phase III Study Testing Efficacy & Safety of Oral Dabigatran Etexilate vs. Warfarin for 6 m Treatment for Acute Symp Venous Thromboembolism (VTE) (RE-COVER II) [25]	NCT00680186								
370,472	Study of Efficacy and Safety of Secukinumab in Patients With Non-radiographic Axial Spondyloarthritis (PREVENT) [26]	NCT02696031	European Union	EPAR	555	1:1:1	1 stratum, 3 levels	6	2	0.36%

^a^%FR is calculated as the number of reported forced randomizations (using the definition that a manual mis-dispensation is not considered a forced randomization) divided by the total number of randomized patients

^b^In addition, this study had 4 manual mis-dispensations that were not included in our calculation of %FR

Overall, only 8 unique trials reported to have used forced randomization. The reported percentage of forced allocations ranged from 0.34 to 7.76%. It should be noted that 7.76% was an outlier among the set of trials; the next highest percentage was 3.30%.

In general, no information was provided on how FR patients were addressed in the analysis; FR were most often counted as protocol deviations. One document described including the FR patients in the efficacy analyses according to their planned treatment (a problematic approach in our view), and for the safety analyses according to the actual treatment (one reviewer document requested that this was done); the safety analysis using “as treated” approach is also standard across industry.

One interesting finding was related to the interpretation of what constitutes a FR. It is very clear that the definition of “forced randomization” related to the dispensation of medication kits as the unit of randomization, i.e., whenever the kit rather than the pre-generated randomization schedule was the driving factor, a patient would be considered as “forced”. This means that there can be different categories of forced randomizations. This was most clearly articulated in one of the documents (IDRAC 304185 in Table 2), where the following three types of “forced randomization” were described:

1. “*Manual randomizations*, [where] *subject could have received the correct treatment allocation per randomization or could have led to incorrect treatment allocation.”*
2. *“Forced randomization* [that occur via] *programmed algorithm to the correct treatment assignment per original randomization scheme.”*
3. *“Forced randomization* [that occur via] *programmed algorithm to the incorrect treatment assignment.*”

Most frequently, however, “forced randomization” related to the definition classified in the quote’s 3rd scenario above.

Given the small number of reports uncovered through the search, it is unclear how representative these trials are with respect to the approaches to forced randomization. Based on conversations with IRT providers, the use of FR is much more common in multi-center trials. The specifications of the randomization procedure, including the details on the randomization method (permuted blocks or another type) and whether the forced randomization is allowed, backfilling is used, the cap on the number of forced randomizations is set, etc., are listed in the IRT specifications document. These details are not included in the protocol to disclose as little as possible to the investigators and thus minimize the potential for selection bias. Considering that the described randomization procedure is followed in the study, it is unclear why the instances of forced randomization should be considered as a deviation from the protocol.

## Planning for the forced randomization option at the trial design stage: The “How”

The need for FR should be carefully assessed at the study design stage. For instance, if drug supply is scarce and the drug cost is high, then instead of using the central (unstratified) randomization schedule, one may consider center-stratified randomization that will obviate the need for FR. If stratification on several prognostic factors is required, a stratified modified Zelen’s approach or some other dynamic allocation procedures that operate with a limited stock on site while providing across study balance in prognostic factors can be used [10, 13]. Alternatively, special designs that are explicitly based on cost-efficiency considerations may be considered [27].

If central randomization is necessary and FR is an acceptable option, care should be taken to mitigate the risk of partial unblinding and risks of other potential biases, as described in Table 1. In addition, the designer of a clinical trial may consider performing Monte Carlo simulations to estimate the expected percentage of forced randomizations and other operating characteristics given the assumptions and the input parameters for the study. In what follows, we describe a structured approach for developing and evaluating different randomization designs with or without FR through simulation.

### Simulation study setup

A simulation protocol is an essential document for planning in silico experiments in drug development [28, 29]. This protocol should capture the key input parameters, clinical trial assumptions, the processes to be simulated, the operating characteristics of interest, the procedures to be compared, and other relevant details.

Throughout, we shall assume that the trial is designed as a randomized, parallel-arm, multi-center study using central randomization (i.e., study participants may be recruited by different study centers but are randomized according to a common randomization sequence that can be pre-generated). In this version, we assume for simplicity that each patient receives a treatment kit just once – at the randomization visit. In other words, once a participant is recruited and randomized, he or she should be immediately supplied with a single kit of randomized treatment (A or B) and subsequently there will be no need for additional drug supply for this participant. Some important parameters for simulation are:

- Sample size ($\:n$) – the total number of patients to be randomized in the study.
- Number of treatment arms – e.g., two-arm study in our example.
- Target treatment allocation ratio – e.g., 1:1 corresponds to a two-arm equal allocation in our example.
- Randomization method to implement (exactly or approximately) the target allocation ratio – e.g., permuted blocks or some other method.
- Patient recruitment process – e.g., Poisson-gamma recruitment model [30], in which case some additional parameters need to be specified:
  Number of study sites/centers ($\:N$).
  Recruitment policy for the sites – e.g., competitive, balanced, or restricted recruitment policy [31].
  Site activation model – e.g., sites are activated over a given time interval.
  Recruitment rates for the sites – e.g., rates constitute a random sample from a gamma distribution with given hyperparameters.

(f) Drug supply chain management parameters:
  Initial supply to the study sites.
  Frequency of evaluation of drug supply levels across the sites – e.g., once a week.
  “Trigger” level – the condition for a current level of drug supply on site under which a re-supply order is made.
  Re-supply policy – how many drug kits should be sent to a site upon request.

(g) Delivery time to the sites – fixed or random (it may also depend on the site geography).
(h) Cost of drug supply and shipment.
(i) Other parameters that may be deemed appropriate for the trial.

The main purpose of a simulation study may be to compare—for a set of chosen trial assumptions—the four selected configurations of the IRT (cf. Fig. 1) in terms of the operating characteristics that may include:

- Treatment imbalance at the time of reaching target recruitment (difference between the number of patients randomized to A and B).
- Proportion of forced allocations during the recruitment period.
- Proportion of patients sent home (not randomized) due to lack of drug supply at study sites (applies to FR0a and FR0b only).
- Number of patients who are waitlisted (this is in a situation where there is no drug available at the site; patients are called to stay at home and allocated once adequate drug supply is available at that site, right after the supply arrives).
- Number of patients who are not allocated (this is the number of patients left on the waiting list once target recruitment is reached). In practice, these patients would most likely be randomized resulting in the actual number of allocated subjects exceeding the planned allocation number. But to have a cleaner comparison of the options, we assumed the randomization is stopped when the target number is achieved.
- Drug supply overage: (total drug – ideal amount)/ideal amount, where ideal amount in the 1:1 example considered in this paper is the amount of drug supply under the (unrealistic) assumption of the distribution of $\:n/2$ kits of each drug type A and B per treatment group (assuming $\:n$ is even).
- Time to complete the recruitment by enrolling and randomizing $\:n$ patients in the study.

### An example

Consider a multi-center 1:1 RCT with two treatments, A and B. The total number of patients to be randomized is $\:n=500$. The randomization method is central (unstratified) permuted block design (PBD) with blocks of fixed length of 4; that is, treatment assignments are generated sequentially in blocks of 4 such that within each block exactly 2 allocations are made at random to each of the treatments A and B. Assume there are $\:N=80$ centers which follow a competitive recruitment policy, i.e., there is no restriction on the number of patients recruited per center, and the recruitment stops once $\:n$ patients have been randomized into the study. The target recruitment period is $\:T=12$ months. Study centers are activated independently, and the activation time for the $\:i$th center is $u_{i}\sim{Uniform(0,4)}$, i.e., it is assumed that all centers are activated during the first 4 months.

Assume a Poisson-gamma process for patient recruitment [30] with a medium mean recruitment rate of 0.525 patients per center per week ($\:m=0.525/7=0.075$ patients per center per day). Then by setting $\:\alpha\:=1.2$ and $\:\beta\:=\frac{\alpha\:}{m}=16$, the recruitment rate for the $ith$ center is $\:{\lambda\:}_{i}\sim\:Gamma\left(\alpha\:,\beta\:\right)=Gamma(1.2,\:16)$. Here ($\:\alpha\:,\:\beta\:$) are the shape and rate parameters of a gamma distribution with probability density function $\:p\left(x|\alpha\:,\beta\:\right)=\frac{{\beta\:}^{\alpha\:}}{{\Gamma\:}\left(\alpha\:\right)}{x}^{\alpha\:-1}{e}^{-\beta\:x}$, $\:x>0$, in which case $\:E{\lambda\:}_{i}=\frac{\alpha\:}{\beta\:}$ and $\:{var\:\lambda\:}_{i}=\frac{\alpha\:}{{\beta\:}^{2}}$. The $\:{\lambda\:}_{i}$’s are independent and identically distributed gamma random variables.

We will assume that for all active centers drug supply levels are evaluated on a weekly basis, at times = 7, 14, … Let $\:{k}_{A,i}\left(t\right)$ be the number of kits of drug A available at the $\:i$th center at time $\:t>0$, and $\:{k}_{B,i}\left(t\right)$ be the similar quantity for drug B. A re-supply order for drug A is made for the $ith$ center, if $\:{k}_{A,i}\left(t\right)\le\:Crit$, where $\:Crit$ is a pre-determined small positive integer that defines the trigger event for drug re-supply.^2^ Likewise, a re-supply order for drug B is made for the $ith$ center, if $\:{k}_{B,i}\left(t\right)\le\:Crit$.

In our simulation study, we will compare the performance of 4 different IRT system configurations (see Fig. 1) and 3 different supply/re-supply strategies—Low, Medium, and High—that are characterized by different levels of initial supply, trigger levels, and re-supply policies (see Table 3).

**Table 3:** Drug re-supply strategies

	Low	Medium	High
**Initial drug supply** ($\:{\varvec{k}}_{\varvec{A},0},{\varvec{k}}_{\varvec{B}0}$)	(2, 2)	(3, 3)	(4, 4)
Trigger level ($\boldsymbol C\boldsymbol r\boldsymbol i\boldsymbol t$)^a^	1	1	2
**Re-supply policy (to achieve the specified levels** ($\:{\varvec{k}}_{\varvec{A}},{\varvec{k}}_{\varvec{B}}$) **of drug onsite)**^b^	(2, 2)	(4, 4)	(5, 5)

^a^A re-supply order for drug $\:d$ ($\:d=A,B$) is made for the $ith$ center, if $\:{k}_{d,i}\left(t\right)\le\:Crit$

^b^A re-supply order is made for the $ith$ center to achieve the specified number of ($\:{k}_{A},{k}_{B}$) kits at the center

With the Low strategy, initially 2 kits of each treatment A and B are supplied to the site upon its activation. The initial numbers of treatment A and B kits per site are (3, 3) for the Medium strategy, and (4, 4) for the High strategy. As the site recruits and randomizes patients, the amount of drug supply available at the site will decrease over time, necessitating a re-supply order. Suppose at time $\:t>0$, $\:{k}_{A,i}\left(t\right)=1$ and $\:{k}_{B,i}\left(t\right)=0$ (one kit of drug A and no kits of drug B are available at the $ith$ site). In this case, with the Low strategy, a re-supply order is made in the amount of 1 kit of drug A and 2 kits of drug B to achieve fixed levels of drug supply $\:{k}_{A}=2$ and $\:{k}_{B}=2$. In the same example ($\:{k}_{A,i}\left(t\right)=1$ and $\:{k}_{B,i}\left(t\right)=0$), the Medium and High strategies would request different amounts of re-supply: the Medium strategy would request 3 kits of drug A and 4 kits of drug B to achieve fixed levels $\:{k}_{A}=4$ and $\:{k}_{B}=4$, whereas the High strategy would request 4 kits of drug A and 5 kits of drug B to achieve fixed levels $\:{k}_{A}=5$ and $\:{k}_{B}=5$ (see Table 3).

#### Results for the “base case” scenario

**Fig. 4:**
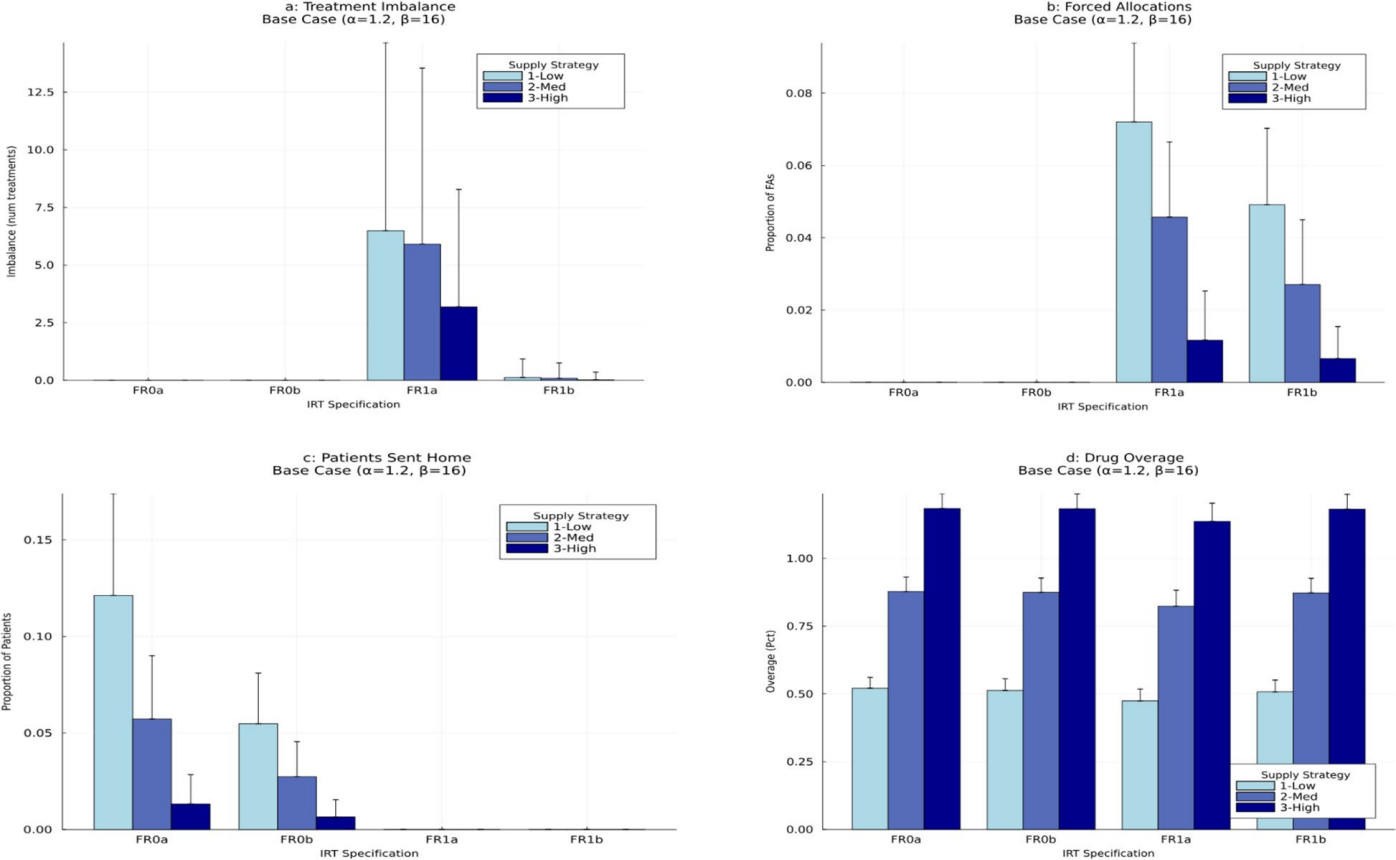
Operating characteristics of 12 randomization approaches under the “base case” scenario The displayed operating characteristics are: final treatment imbalance (top left plot); proportion of forced allocations (top right plot); proportion of patients not randomized due to drug supply shortage (bottom left plot); and drug overage (bottom right plot). The height of each bar (rectangle) is the simulated mean value of the measure of interest. Error bars are added to quantify uncertainty. The width of each error bar is equal to 1.645*SD, where SD is the simulated standard deviation of the measure of interest

**Fig. 5:**
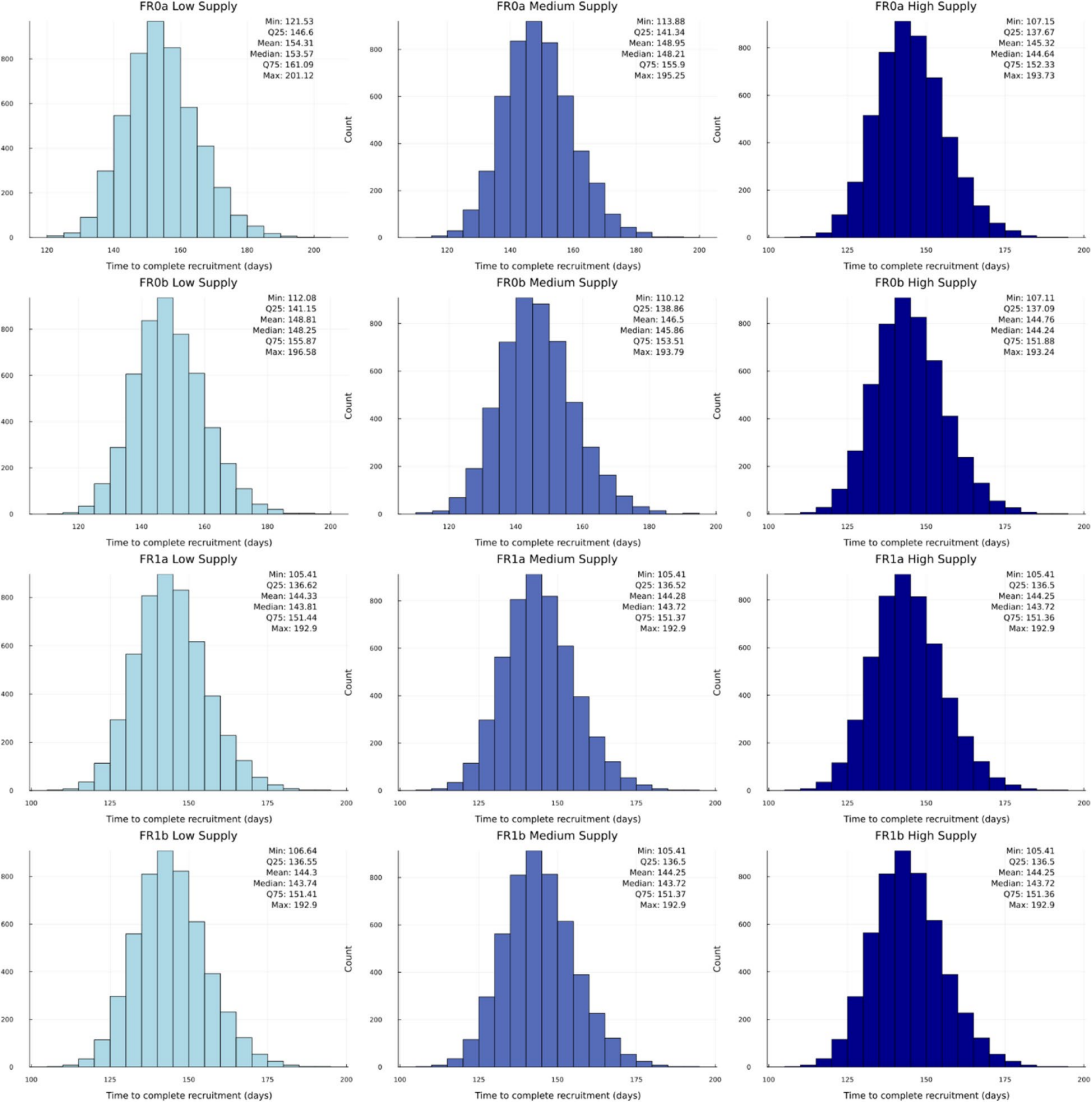
Histograms of simulated distributions of time to complete recruitment of 12 randomization approaches in the “base case” scenario

For the described experimental setting, which we refer to as the “base case” scenario, we simulated patient enrollment and randomization under 12 different approaches – i.e., 12 combinations of an IRT configuration (FR0a, FR0b, FR1a, FR1b) in combination with a re-supply strategy (Low, Medium, High). For each considered approach, a trial was simulated 5,000 times. The design operating characteristics are summarized in Table S1 in the Supplemental Appendix. The key results are displayed in Figs. 4 and 5 below.

Figure 4a shows the mean with associated uncertainty (quantified by a 90% upper confidence limit, UCL, defined as 1.645*SD, where SD is the simulated standard deviation) of treatment imbalance for the 12 considered approaches. When FR was not allowed (FR0a or FR0b), then irrespective of the resupply strategy (Low, Medium, or High), the final treatment numbers were always balanced (cf. first six categories in Fig. 4a) since the randomization schedule was strictly followed. For FR without backfilling (FR1a), the final imbalance could be non-zero (on average, at most ~ 6.4; categories 7, 8, 9 in Fig. 4a). However, when FR with backfilling was used (FR1b), the imbalance was nearly zero, irrespective of the re-supply strategy (cf. categories 10, 11, 12 in Fig. 4a).

Figure 4b displays the mean (90%UCL) of proportion of forced allocations for the 12 approaches. This proportion was equal to 0 in situations when FR was not allowed (FR0a or FR0b) (cf. first six categories in Fig. 4b). For FR1a and FR1b (cf. categories 7–12 in Fig. 4b), the average proportion of forced allocations was small but non-zero. It was ~ 7% for FR1a with Low re-supply strategy, and it was ~ 5% for FR1b with Low re-supply strategy. At the same time, with High re-supply strategy, this proportion was ~ 1% for both FR1a and FR1b. Therefore, increasing the intensity of drug re-supply from Low to High helped significantly reduce the need for forced allocations.

Figure 4c shows the mean (90%UCL) of proportion of patients sent home (not randomized due to a lack of drug supply on site) for the 12 approaches. This proportion was calculated relative to the targeted sample size ($\:n=500$). In our considered setting, this proportion was highest (on average, ~ 12%) for FR0a with Low re-supply strategy. At the same time, with High re-supply strategy, this proportion was, on average, ~ 1% for FR0a and FR0b. Using configurations with FR allowed (FR1a or FR1b) in combination with any re-supply strategy helped ensure that the proportion of patients sent home was zero (cf. categories 7–12 in Fig. 4c).

Figure 4d shows the mean (90%UCL) of drug overage for the 12 approaches. One can see that the drug overage depends mainly on the re-supply strategy – Low, Medium, or High – and to a lesser extent on the IRT configuration. As expected, the overage was lowest (~ 47–52% higher than the “ideal”) with the Low re-supply strategy; it was ~ 82–88% higher than the “ideal” with the Medium re-supply strategy; and it was ~ 114–118% higher than the “ideal” with the High re-supply strategy. For any given re-supply strategy, FR1a had a slightly lower overage compared to the other IRT configurations, which may be due to that FR1a came along with the highest percentage of forced allocations.

We also obtained the summaries of the number of patients who were waitlisted and the number of patients who were not allocated (remained on the waiting list at the time when the recruitment was completed) with the 12 considered approaches. With FR0a, the number of patients waitlisted and not allocated was exactly zero for all re-supply strategies. With FR0b and Low re-supply strategy, on average, there were about 6 patients waitlisted and 0.1 patient not allocated. Using a High re-supply strategy reduced these numbers to near zero. With FR enabled, the average numbers of patients who were waitlisted and not allocated increased: with FR1a and Low re-supply strategy, about 20 patients were waitlisted and 0.6 patients not allocated; with FR1b and Low re-supply strategy, there were 13 patients waitlisted and 0.4 patients not allocated. Changing the re-supply strategy to High resulted in < 1 patient waitlisted and < 0.03 patients not allocated with both FR1a and FR1b.

Figure 5 displays the histograms of the simulated distribution of total time to complete study recruitment for the 12 approaches. Overall, the shapes of the histograms are very similar. In our considered experimental setting, it took, on average, between 144 and 154 days to enroll and randomize 500 patients with the 12 approaches. The largest average time (154 days) was for FR0a with Low re-supply strategy which is consistent with the highest percentage of subjects refused randomization. The similar quantity for forced randomization with backfilling (FR1b) and High re-supply strategy was ~ 144 days.

#### Results for the “slower recruitment” scenario

We performed additional simulations to investigate the sensitivity of the results to change in trial assumptions. The “slower recruitment” scenario had the mean recruitment rate of 0.3 patients per center per week, i.e., ~ 43% slower recruitment compared to the “base case” scenario of 0.525 patients per center per week, which corresponded to a Poisson-gamma recruitment model with $\lambda_{i}\sim Gamma({1.2,\:28})$ instead of $\:Gamma(1.2,\:16)$. The operating characteristics under the “slower recruitment” scenario are summarized in Table S2 in the Supplemental Appendix, and the key findings are highlighted below:

- Treatment imbalance was zero for FR0a and FR0b; it was small but not zero (and slightly reduced compared to the “base case” scenario) for FR1a; and it was negligibly small (on average, < 1) for FR1b with any re-supply strategy.
- The proportion of forced allocations was zero for FR0a and FR0b; it was small but not zero for FR1a with Low re-supply strategy (e.g., ~ 5% under “slower recruitment” vs. ~7% under “base case”); and it was at most 2% for FR1b.
- The proportion of patients sent home was zero for FR1a or FR1b in combination with any re-supply strategy. For FR0a and FR0b, this proportion was small but not zero (e.g., for FR0a with Low re-supply strategy, it was ~ 5% under “slower recruitment” vs. ~12% under “base case”).
- The drug overage results were, overall, consistent with those under “base case” scenario. The results depended mainly on the re-supply strategy but not on the IRT configuration. The overage was ~ 50–55% higher than the “ideal” with the Low re-supply strategy; it was ~ 84–90% higher than the “ideal” with Medium re-supply strategy; and it was ~ 118–121% higher than the “ideal” with High re-supply strategy.
- The number of patients waitlisted, and the number of patients not allocated were smaller than those under “base case” scenario. With High re-supply strategy, these numbers were (nearly) zero for all four IRT configurations.
- The time to complete recruitment, on average, was ~ 207–215 days under “slower recruitment” (vs.~144–154 days under “base case”).

#### Results for the “faster recruitment” scenario

The “faster recruitment” scenario assumed the mean recruitment rate of 1.68 patients per center per week, i.e., 3.2 times higher rate compared to the “base case” scenario of 0.525 patients per center per week, which corresponded to a Poisson-gamma recruitment model with $\lambda_i\sim Gamma(1.2,\:5)$ instead of $\:Gamma(1.2,\:16)$. The operating characteristics under the “faster recruitment” scenario are summarized in Table S3 in the Supplemental Appendix, and the key findings are highlighted below:

- Treatment imbalance was zero for FR0a and FR0b; it was small but not zero (and slightly higher than in the “base case” scenario) for FR1a; and it was still negligibly small (on average, < 1) for FR1b with any re-supply strategy.
- The proportion of forced allocations was zero for FR0a and FR0b; however, it was slightly increased compared to the “base case” for FR1a and FR1b. Under “faster recruitment”, the proportion of forced allocations was, on average, between ~ 5% (FR1a or FR1b used in combination with High re-supply strategy) and ~ 12% (FR1a or FR1b used in combination with Low re-supply strategy).
- The proportion of patients sent home was zero for FR1a or FR1b in combination with any re-supply strategy. For FR0a and FR0b this proportion was increased compared to the “base case” scenario. For instance, FR0a with Low re-supply strategy resulted, on average, in ~ 52% of the targeted number of 500 patients sent home due to the lack of drug supply. Using FR0a or FR0b with High Supply strategy helped reduce this number to ~ 12% and ~ 6%, respectively. These numbers may be still regarded as high, indicating that an adjustment in re-supply strategy (increased intensity) may be warranted.
- The drug overage results were, overall, consistent with those under “base case” scenario. The drug overage was primarily determined by the re-supply strategy, and to a lesser extent on the IRT configuration. The overage was ~ 46–49% higher than the “ideal” with Low re-supply strategy; it was ~ 83–87% higher than the “ideal” with Medium re-supply strategy; and it was ~ 111–115% higher than the “ideal” with High re-supply strategy.
- The number of patients waitlisted, and the number of patients not allocated increased substantially across the board, besides when FR0 was used, where it stayed at exactly 0. For instance, ~ 79 patients were waitlisted and ~ 7 patients were left not allocated, on average, when using FR0a with Low re-supply strategy. Overall, the largest values for number of patients waitlisted and not allocated were with FR1a in combination with Low re-supply strategy, with ~ 134 patients waitlisted and ~ 15 patients left waiting (not allocated), on average. The lowest values were with FR0b in combination with High re-supply strategy, where ~ 9 patients were waitlisted, and ~ 0.4 patients were left waiting (not allocated), on average.
- The time to complete recruitment, on average, was ~ 75–95 days under “faster recruitment” (vs. 144–154 days under “base case”). The longest average time (95 days) was for FR0a with Low re-supply strategy. The similar quantity for FR1b and High re-supply strategy was ~ 74 days.

### Additional simulations

To get further insights into statistical properties of IRT configurations and re-supply strategies, we ran a simulation study assuming that a smaller number of study sites ($\:N=16$) is used to recruit a smaller number of patients ($\:n=100$). All other parameters (target trial duration, site activation pattern, Poisson-gamma model for patient recruitment, supply/re-supply levels) were kept the same. The detailed summaries of simulation results under three scenarios for the recruitment rate of patients per site per day ($\:m$) – “base case” ($\:m=0.075$), “slower recruitment” ($\:m=0.0429$), and “faster recruitment” ($\:m=0.24$) – are presented in Tables S4–S6 in the Supplemental Appendix. Overall, the results of these additional simulations with $\:N=16$ and $\:n=100$ were consistent in terms of time to complete recruitment and drug overage, as compared with the original simulations with $\:N=80$ and $\:n=500$. The numbers for imbalance, proportion of forced allocations, and number of patients waitlisted/not allocated tended to be smaller in the additional simulations compared to the original simulations,

Further simulations can be easily performed using our developed Julia code, which is available at https://github.com/csch7/forced-randomization.

## Conclusions

### Summary and discussion

In this paper, we investigated the phenomenon of forced randomization (FR), an approach for implementing randomized treatment assignments in the presence of some restrictions related to drug supply management in multi-center RCTs with central randomization handled by interactive response technology (IRT). When used properly, FR can help improve the logistics, drug supply management, duration of the enrollment and the cost-efficiency of the RCT – which may be particularly important in studies with expensive drug and/or in situations when the drug supply is scarce. At the same time, FR carries some potential risks that should be carefully reviewed at the study planning stage, and, ideally, prospectively addressed through risk mitigation planning.

The planning of FR should be considered jointly with other components of a multi-center RCT, such as the patient recruitment process and the drug supply management model [30–35]. For understanding statistical properties of FR, Monte Carlo simulation studies can be useful and may be the only feasible option to obtain the operating characteristics for such complex processes [36]. We provided an example of applying our framework in a hypothetical multi-center 1:1 RCT with 500 patients. Our goal was to explore the effect of four different IRT configurations in combination with three different drug supply/re-supply strategies on some important operating characteristics of the trial. Our key assumptions for the “base case” scenario concerned the recruitment process (a Poisson-gamma model with 80 centers that were activated independently over a 4-month period and followed a competitive recruitment policy to enroll 500 patients in the target period of 12 months, with an average recruitment rate of 0.525 patients per center per week), the drug supply management model (weekly inspection of drug supply levels across the sites, three different choices of the intensity of drug re-supply – Low, Medium, and High, a fixed delivery time of 2 days from the central drug depository to any study center), and the randomization method (central, unstratified 1:1 randomization implemented using permuted blocks of 4).

Table 4 summarizes our key findings from the simulations, which can aid the reader to decide on what type of IRT configuration may be best suited for their trial, taking into consideration the recruitment speed and the proposed supply strategy.

**Table 4.**
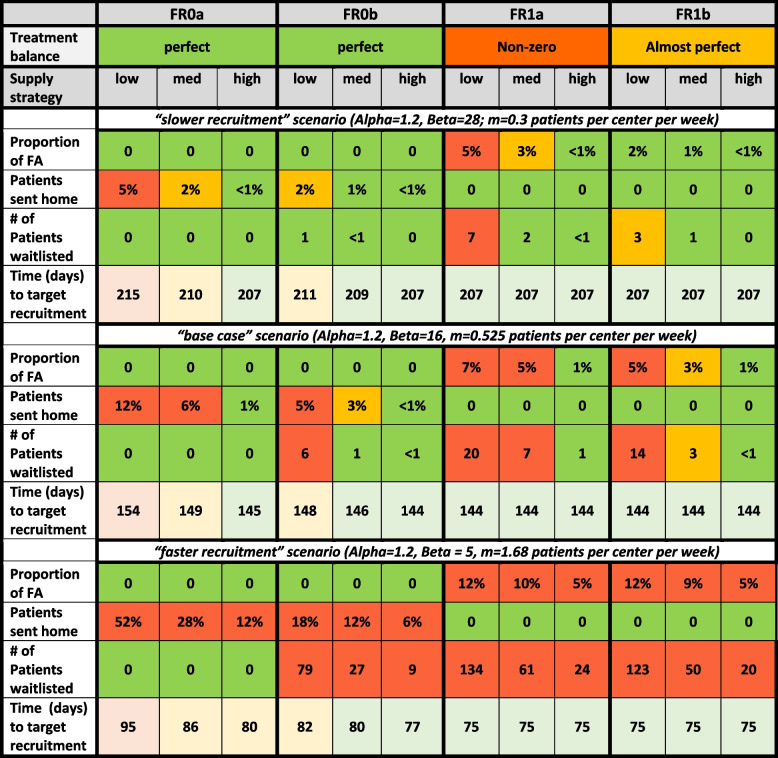
Decision table based on the key simulation findings under the considered scenarios

“Non-zero” balance for FR1a refers to the possibility of observing a quite significant treatment imbalance depending on the number of skipped randomization numbers on the list that are not backfilled (thereby closing the gap towards complete balance). “Almost perfect” balance for FR1b can be observed despite back-filling depending on the resupply levels at other sites. However, due to the backfilling option this will be markedly lower than the FR1a option

Under the considered “base case” scenario for enrollment and the supply/re-supply strategies, the percentage of forced allocations is expected to be in the range of 1–7% for FR1a and FR1b. The treatment imbalance at the end of the study is small but non-zero when backfilling is not used (FR1a), and it is negligible when backfilling is used (FR1b). By using FR with or without backfilling, one can eliminate the chance of an undesirable event of not enrolling eligible patients into the study due to a lack of drug supply on site. As regards drug overage, we found that it is primarily determined by the intensity of the re-supply strategy, and to a much smaller extent by the presence or absence of the FR feature in the IRT. Finally, the High re-supply strategy enables some reduction (~ 8–11 days, on average) in the time to complete the target enrollment of patients, regardless of what FR option is chosen. For configurations that allow forced randomization, such reduction is mainly achieved by eliminating situations when the site has no drug at all while patients are already available for randomization at the site. It should be noted that while the simulations assumed the patients sent home after coming to the site for randomization will return for randomization when the drug re-supply arrives, in real life this negative experience might make the patient to change their mind about participation in a trial. With that, more patients will need to be screened and an impact on the duration of enrollment might be even higher.

Overall, under the “base case” setup, using FR with a carefully chosen supply/re-supply strategy can result in quantifiable improvements in the trial logistics and efficiency.

Clearly, the described findings depend on various assumptions, and investigating the sensitivity of the results to changes in the input parameters is very important. We did two such sensitivity assessments (referred to as “slower recruitment” and “faster recruitment”) by changing an assumption on the mean recruitment rate – decreasing it by 43% or increasing it 3.2-fold compared to the “base case” scenario – and keeping all other parameters unchanged.

Under the “slower recruitment” scenario, the proportion of forced allocation slightly decreased for FR1a and FR1b configurations compared to the “base case”. With backfilling (FR1b), irrespective of the re-supply strategy, the final imbalance was negligibly small. The drug overage results were consistent with those under the “base case” scenario. As expected, “slower recruitment” led to significant increase in the time to complete the target enrollment of patients.

Under the “faster recruitment” scenario, the proportion of forced allocation increased, albeit it was still relatively small (~ 5–12% depending on the IRT configuration (FR1a, FR1b) and the re-supply strategy). With backfilling (FR1b), the final imbalance was still negligibly small, and the drug overage results were consistent with those under the “base case” scenario. “Faster recruitment” led to significant reduction in the time to complete the target enrollment of patients that varied between IRT strategies, greatly favoring the options with or without backfilling turned on (FR1a, FR1b).

Overall, based on our simulation evidence, forced randomization with backfilling (FR1b) in combination with High re-supply strategy seems to be the optimal choice for the considered experimental settings.

In practice, a trialist is often asked to define a “cap” when including FR in the IRT. This cap reflects the number of patients that the trialist will be comfortable with to be forced randomized during the recruitment period of the trial. Once this cap is reached, the IRT system will no longer allow additional forced randomizations. For example, our simulations show that for the “base case” recruitment scenario, a cap of ~ 10% of the overall sample size would not interfere with randomization in 90 or more percent of cases for FR1a option with Low re-supply strategy, whereas a cap of 2.5% would suffice for the same purpose when considering FR1a option with High re-supply strategy (see Table S1 in Supplemental Appendix). However, if the recruitment is slower or faster, the value of the cap needs to be adjusted. Likewise, if we consider using FR1b option, the “optimal” value of the cap will depend on the recruitment speed, re-supply strategy, and possibly other parameters of the trial.

During this research the authors frequently discussed the purpose of choosing and setting a cap in the hopes of providing the reader with a helpful strategy. In our opinion, setting the cap does not solve the problem of what to do in case of drug shortages, but just postpones it to a later moment in the trial if the drug supply strategy does not support the cap. If the cap is reached in the middle of the trial, it means that the randomization strategy is different between the first part of the trial and the rest of the trial, which is also undesirable. Overall, we think that rather than setting the cap on the number of forced allocations, an alert should be set at a small number of forced allocations for the drug manager to investigate the cause and evaluate whether there are other mitigation factors that could be put in place to reduce further forced randomizations (e.g., by improving the supply strategy, re-enforcing sites in acknowledging the receipt of the supplies, better supporting the sites with high enrollment, etc.).

When using forced randomization, we recommend the backfilling of skipped allocations on the randomization schedule. As the simulations show, this approach leads to a better balance in treatment assignments at the end of the trial and smaller percentage of forced allocations in considered examples. Additionally, if some sites experience a temporary shortage of one drug (for example, drug A) during a certain time period, the backfilling will allow other sites that have the drug available to backfill the skipped A assignments and maintain balance in treatment assignments in time. Without backfilling, there will be more allocations to drug B than to drug A during this time period and the accumulated imbalance in treatment assignments will never be recovered. This could also lead to an accidental bias if a time trend in outcomes is present. A massive stockout when all sites experience absence of the same drug should be avoided with any randomization strategy as it could lead to biased results.

A great advantage of FR is that the sites and the study team remain unaware of the fact that forced allocations took place and thus remain properly blinded. The notifications that forced allocations took place for specific subjects should only go to the unblinded drug supplies managers, who will not communicate it to the study team. To better conceal the instances of forced allocations it is recommended to use scrambled allocation numbers, since otherwise allocation numbers out of chronological order of randomization might indicate a forced allocation. If the trial is open-label, caution may be warranted to ensure that FR is not influenced by other factors, such as using FR to “assign” patients with better prognosis to the experimental treatment arm, e.g., by observing that only one drug type is present at the site, or even by purposefully destroying treatment kits labeled as the control arm. It should be acknowledged that varying other assumptions on the patient recruitment process and the drug supply chain parameters (e.g., the frequency of inspection of drug supply, the amount of initial supply/re-supply, the trigger level, the time to deliver the drug to the site, etc.) may lead to some further useful findings and may supplement the ones obtained under the considered experimental settings. Our code for simulations is fully documented and can be used for this purpose (and it is available upon reasonable request).

### Limitations and future work

While our simulation study has provided many useful insights into the phenomenon of FR, it has some limitations. We focused on 1:1 RCT; however, modern clinical trials are increasingly designed as multi-arm clinical trials with possibly unequal allocation ratios. We perceive that a general approach to FR issues in multi-arm trials should be similar to the 1:1 RCT case, but there will be some nuances related to the choice of a randomization method, e.g., permuted block design vs. some novel approaches such as brick tunnel randomization [37], block urn design [38], mass weighted urn design [39], etc. The supply/re-supply strategy would have to be fine-tuned depending on the number of arms, treatment allocation ratio, and other factors. With some complex trial designs such as platform trials [40], the considerations become more intricate as the number of treatment arms and the allocation ratio can be modified adaptively in a platform trial.

In our current study, we assumed that only one drug kit is required for each study participant, while in practice many studies use repeated dosing schedules, and there is a requirement that the drug is dispensed multiple times, beyond the “loading dose”, for each trial participant. The expiry of drug supply, potential drug kit damages and drug dispensing errors may be additional important aspects that need to be accounted for and managed throughout the trial.

In the simulations, we assumed that the drug re-supplies are triggered automatically based on the re-supply policy. In practice, a lot of manual monitoring is often involved in the request of the re-supplies: for example, the number of subjects in screening is considered, or the institutions with high-volume enrollment are given higher level of resupplies and closer monitoring. This type of careful and conscientious monitoring is likely to reduce the rates of FR compared to our simulations, but the general conclusions from the simulations remain valid.

We assumed that there is a central depot that contains a very large amount of drug supply, sufficient to cover the demand for the given trial. While this may be a reasonable assumption in some circumstances (e.g., low molecular weight pharmaceutical products), this may not be the case for other advanced treatment modalities such as CAR T-cell therapies [41].

Another important consideration not covered in the present work is the evaluation of statistical properties of randomization designs featuring FR. Reference [15] provides some simulation evidence showing that the type I error rate of a two-sample t-test may be minimally affected (i.e., procedures are slightly conservative) in the presence of a linear trend in the outcome if FR with backfilling is used. For the considered randomization procedures, it was found that the addition of FR preserves the properties of the original procedure with respect to the Type I error in presence of the linear trend. A more in-depth study will be pursued in the upcoming work. A question of whether there is a need to account for the cases of forced allocation in the analysis – e.g., performing a primary ITT efficacy analysis according to the actual treatment assignment, and performing a sensitivity analysis of some kind – is an important open problem that merits investigation. If the population model is followed in the analysis, the randomization mechanism is irrelevant and the ITT analysis, where patients are analyzed according to the treatment group they were randomized to, is appropriate [2].

A robust alternative to standard statistical tests based on the assumption of the population (random sampling) model is the randomization-based inference or the so-called “re-randomization tests” [2]. With FR, the construction of re-randomization tests may be complicated – one would need to know the full history of randomized treatment assignments and the drug supply availability at each study site throughout the trial. However, what is available at a site is also impacted by the treatment assignments given to the patients at the site (and, also, other sites). Thus, a drug supply/resupply algorithm would be needed to construct a reference set to perform a re-randomization test (and taking care of the manual adjustments to the algorithm does not seem feasible). Overall, the re-randomization test following forced allocation does not seem feasible and the practical need for such analysis is also unclear.

In summary, forced randomization can be a useful allocation option to consider in practice, that can, when properly planned for and carefully implemented, reduce patient burden, and enable faster recruitment times. We hope to address some of the important problems outlined above in the future work.

## Supplementary Information

Supplementary Material 1.

## Data Availability

All results reported in this paper are based either on theoretical considerations or simulation evidence. The computer code (using Julia programming language) is fully documented and is available at https://github.com/csch7/forced-randomization.

## Footnotes

1 Throughout the paper, we will use the terms “forced allocation” and “forced randomization” interchangeably, acknowledging that forced allocation refers to an individual treatment assignment accounting for the availability of drug supply at the site, whereas forced randomization may refer to an individual treatment assignment and, more broadly, the method of performing such treatment assignments.

2 In practice, the choice of the value of $\:Crit$ will depend on the trial assumptions/parameters such as the site recruitment rates, the drug delivery time to the sites, etc. For any specific trial it can be determined using simulation setup described above with input from the clinical trial management team, drug supply management, study investigators, and other relevant stakeholders.

## Abbreviations

IVD: In vitro diagnostics
EPAR: European public assessment report
BLA: Biologic License Application
NDA: New Drug Application
