# Supplementary material for: Forced randomization: the what, why, and how
Source: BMC Med Res Methodol. 2024 Oct 8;24:234. doi: 10.1186/s12874-024-02340-0 (PMC11459895; doi:10.1186/s12874-024-02340-0)
Supplement: Supplementary file 1 — Supplementary Material 1. [file 12874_2024_2340_MOESM1_ESM.pdf]

Table S1: Summary statistics - Mean (SD) [Q90] - of design operating characteristics under the "base case" experimental scenario (with mean recruitment rate of 0.525 patients per center per week, which corresponds to a Poisson-gamma model with $\lambda\_i \sim Gamma(1.2,16)$), for 12 different combinations of an IRT configuration (FR0a, FR0b, FR1a, FR1b) and a re-supply strategy (Low, Medium, High), with N=80 centers and n=500 patients.

**IRT configuration**	**FR0a**			**FR0b**			**FR1a**			**FR1b**		
**Re-supply strategy**	**Low**	**Medium**	**High**	**Low**	**Medium**	**High**	**Low**	**Medium**	**High**	**Low**	**Medium**	**High**
Imbalance, |D|	0 (0) [0]	0 (0) [0]	0 (0) [0]	0 (0) [0]	0 (0) [0]	0 (0) [0]	6.4 (4.9) [14.5]	6.1 (4.7) [13.8]	3.1 (3.1) [8.2]	0.14 (0.5) [1.0]	0.07 (0.4) [0.7]	0.02 (0.2) [0.4]
Proportion of forced allocations	0 (0) [0]	0 (0) [0]	0 (0) [0]	0 (0) [0]	0 (0) [0]	0 (0) [0]	0.07 (0.01) [0.09]	0.05 (0.01) [0.07]	0.01 (0.01) [0.03]	0.05 (0.01) [0.07]	0.03 (0.01) [0.05]	0.01 (0.01) [0.02]
Proportion of patients sent home[1]	0.12 (0.03) [0.17]	0.06 (0.02) [0.09]	0.01 (0.01) [0.03]	0.05 (0.02) [0.08]	0.03 (0.01) [0.05]	0.01 (0.01) [0.02]	0 (0) [0]	0 (0) [0]	0 (0) [0]	0 (0) [0]	0 (0) [0]	0 (0) [0]
Drug overage	0.52 (0.02) [0.56]	0.88 (0.03) [0.93]	1.18 (0.03) [1.24]	0.51 (0.02) [0.55]	0.88 (0.03) [0.93]	1.18 (0.03) [1.24]	0.47 (0.03) [0.52]	0.82 (0.04) [0.88]	1.14 (0.04) [1.20]	0.51 (0.03) [0.55]	0.87 (0.03) [0.93]	1.18 (0.03) [1.24]
Number of patients waitlisted[2]	0 (0) [0]	0 (0) [0]	0 (0) [0]	6.1 (5.5) [15.1]	1.4 (1.2) [4.7]	0.2 (0.6) [1.1]	20.3 (10.8) [38.0]	7.1 (4.9) [15.2]	0.9 (1.6) [3.6]	13.5 (9.9) [29.7]	3.2 (3.4) [8.8]	0.4 (1.1) [2.3]
Number of patients not allocated[3]	0 (0) [0]	0 (0) [0]	0 (0) [0]	0.1 (0.52) [0.97]	0.03 (0.25) [0.45]	0.00 (0.05) [0.09]	0.60 (1.28) [2.70]	0.22 (0.64) [1.28]	0.03 (0.22) [0.38]	0.39 (1.06) [2.14]	0.07 (0.37) [0.67]	0.01 (0.09) [0.15]
Time (days) to complete recruitment	154 (11) [172]	149 (11) [167]	145 (11) [164]	148 (11) [167]	146 (11) [165]	144 (11) [163]	144 (11) [163]	144 (11) [163]	144 (11) [163]	144 (11) [163]	144 (11) [163]	144 (11) [163]

[1] An eligible patient is sent home (not randomized), if there is a lack of a given drug supply at the study site (applies to FR0a and FR0b only).

[2] In a situation when there is no drug available at the site, a patient is called to stay at home, and they are subsequently allocated to treatment once the drug supply arrives to the site.

[3] Number of patients left on the waiting list once the target recruitment is reached.

Table S2: Summary statistics - Mean (SD) [Q90] - of design operating characteristics under the "slower recruitment" scenario (with mean recruitment rate of 0.3 patients per center per week, which corresponds to a Poisson-gamma model with λ_i~Gamma(1.2,28)), for 12 different combinations of an IRT configuration (FR0a, FR0b, FR1a, FR1b) and a re-supply strategy (Low, Medium, High), with N=80 centers and n=500 patients.

**IRT configuration**	**FR0a**			**FR0b**			**FR1a**			**FR1b**		
**Re-supply strategy**	**Low**	**Medium**	**High**	**Low**	**Medium**	**High**	**Low**	**Medium**	**High**	**Low**	**Medium**	**High**
Imbalance, |D|	0 (0) [0]	0 (0) [0]	0 (0) [0]	0 (0) [0]	0 (0) [0]	0 (0) [0]	5.6 (4.4) [12.8]	4.9 (4.1) [11.7]	1.3 (2.1) [4.7]	0.07 (0.4) [0.7]	0.03 (0.3) [0.4]	0.00 (0.1) [0.2]
Proportion of forced allocations	0 (0) [0]	0 (0) [0]	0 (0) [0]	0 (0) [0]	0 (0) [0]	0 (0) [0]	0.05 (0.01) [0.07]	0.03 (0.01) [0.05]	0.00 (0.01) [0.01]	0.02 (0.01) [0.04]	0.01 (0.01) [0.02]	0.00 (0.00) [0.01]
Proportion of patients sent home[1]	0.05 (0.02) [0.08]	0.02 (0.01) [0.04]	0.00 (0.00) [0.01]	0.02 (0.01) [0.04]	0.01 (0.01) [0.02]	0.00 (0.00) [0.01]	0 (0) [0]	0 (0) [0]	0 (0) [0]	0 (0) [0]	0 (0) [0]	0 (0) [0]
Drug overage	0.55 (0.02) [0.58]	0.90 (0.03) [0.94]	1.21 (0.03) [1.25]	0.55 (0.02) [0.58]	0.90 (0.03) [0.94]	1.21 (0.03) [1.25]	0.50 (0.02) [0.54]	0.84 (0.03) [0.89]	1.18 (0.04) [1.25]	0.55 (0.02) [0.58]	0.89 (0.03) [0.94]	1.21 (0.03) [1.25]
Number of patients waitlisted[2]	0 (0) [0]	0 (0) [0]	0 (0) [0]	1.3 (1.8) [4.1]	0.2 (0.7) [1.4]	0.02 (0.2) [0.3]	6.7 (4.4) [13.7]	2.1 (2.1) [5.6]	0.1 (0.5) [0.8]	3.0 (3.1) [7.8]	0.6 (1.1) [2.4]	0.04 (0.3) [0.5]
Number of patients not allocated[3]	0 (0) [0]	0 (0) [0]	0 (0) [0]	0.02 (0.16) [0.28]	0.00 (0.07) [0.10]	0.00 (0.03) [0.05]	0.11 (0.41) [0.88]	0.04 (0.25) [0.49]	0.00 (0.06) [0.08]	0.03 (0.22) [0.39]	0.01 (0.11) [0.16]	0.00 (0.04) [0.05]
Time (days) to complete recruitment	215 (17) [244]	210 (18) [239]	207 (18) [237]	211 (18) [240]	209 (18) [238]	207 (18) [237]	207 (18) [236]	207 (18) [236]	207 (18) [236]	207 (18) [236]	207 (18) [236]	207 (18) [236]

[1] An eligible patient is sent home (not randomized), if there is a lack of a given drug supply at the study site (applies to FR0a and FR0b only).

[2] In a situation when there is no drug available at the site, a patient is called to stay at home, and they are subsequently allocated to treatment once the drug supply arrives to the site.

[3] Number of patients left on the waiting list once the target recruitment is reached.

Table S3: Summary statistics - Mean (SD) [Q90] - of design operating characteristics under the "faster recruitment" scenario (with mean recruitment rate of 1.68 patients per center per week, which corresponds to a Poisson-gamma model with λ_i~Gamma(1.2,5)), for 12 different combinations of an IRT configuration (FR0a, FR0b, FR1a, FR1b) and a re-supply strategy (Low, Medium, High) with N=80 centers and n=500 patients.

**IRT configuration**	**FR0a**			**FR0b**			**FR1a**			**FR1b**		
**Re-supply strategy**	**Low**	**Medium**	**High**	**Low**	**Medium**	**High**	**Low**	**Medium**	**High**	**Low**	**Medium**	**High**
Imbalance, |D|	0 (0) [0]	0 (0) [0]	0 (0) [0]	0 (0) [0]	0 (0) [0]	0 (0) [0]	6.8 (5.2) [15.3]	7.8 (6.0) [17.7]	6.7 (5.3) [15.4]	0.30 (0.75) [1.5]	0.27 (0.73) [1.5]	0.15 (0.55) [1.1]
Proportion of forced allocations	0 (0) [0]	0 (0) [0]	0 (0) [0]	0 (0) [0]	0 (0) [0]	0 (0) [0]	0.12 (0.02) [0.15]	0.10 (0.02) [0.12]	0.05 (0.02) [0.08]	0.12 (0.02) [0.14]	0.09 (0.02) [0.13]	0.05 (0.02) [0.08]
Proportion of patients sent home[1]	0.52 (0.11) [0.70]	0.28 (0.07) [0.40]	0.12 (0.05) [0.20]	0.18 (0.03) [0.23]	0.12 (0.03) [0.17]	0.06 (0.02) [0.09]	0 (0) [0]	0 (0) [0]	0 (0) [0]	0 (0) [0]	0 (0) [0]	0 (0) [0]
Drug overage	0.49 (0.04) [0.55]	0.87 (0.04) [0.94]	1.15 (0.05) [1.23]	0.47 (0.04) [0.53]	0.86 (0.04) [0.93]	1.14 (0.05) [1.22]	0.46 (0.04) [0.52]	0.83 (0.05) [0.91]	1.12 (0.05) [1.20]	0.47 (0.04) [0.53]	0.86 (0.05) [0.93]	1.14 (0.05) [1.22]
Number of patients waitlisted[2]	0 (0) [0]	0 (0) [0]	0 (0) [0]	79 (29) [126]	27 (18) [56]	9 (10) [26]	134 (34) [189]	61 (26) [104]	24 (17) [52]	123 (34) [180]	50 (26) [92]	20 (17) [47]
Number of patients not allocated[3]	0 (0) [0]	0 (0) [0]	0 (0) [0]	6.8 (7.6) [19.3]	1.2 (2.7) [5.7]	0.4 (1.3) [2.4]	14.8 (12.1) [34.7]	3.3 (4.9) [11.2]	1.2 (2.7) [5.7]	14.4 (11.8) [33.9]	2.7 (4.4) [10.0]	0.95 (2.4) [4.9]
Time (days) to complete recruitment	95 (7) [107]	86 (8) [99]	80 (8) [93]	82 (9) [96]	80 (9) [94]	77 (9) [91]	76 (9) [90]	75 (9) [89]	75 (9) [89]	76 (9) [90]	75 (9) [89]	75 (9) [89]

[1] An eligible patient is sent home (not randomized), if there is a lack of a given drug supply at the study site (applies to FR0 and FR1 only).

[2] In a situation when there is no drug available at the site, a patient is called to stay at home, and they are subsequently allocated to treatment once the drug supply arrives to the site.

[3] Number of patients left on the waiting list once the target recruitment is reached.

Table S4: Summary statistics - Mean (SD) [Q90] - of design operating characteristics under the "base case" experimental scenario (with mean recruitment rate of 0.525 patients per center per week, which corresponds to a Poisson-gamma model with λ_i~Gamma(1.2,16)), for 12 different combinations of an IRT configuration (FR0a, FR0b, FR1a, FR1b) and a re-supply strategy (Low, Medium, High), with N=16 centers and n=100 patients.

IRT configuration	FR0a			FR0b			FR1a			FR1b		
**Re-supply strategy**	**Low**	**Medium**	**High**	**Low**	**Medium**	**High**	**Low**	**Medium**	**High**	**Low**	**Medium**	**High**
Imbalance, |D|	0 (0) [0]	0 (0) [0]	0 (0) [0]	0 (0) [0]	0 (0) [0]	0 (0) [0]	2.9 (2.1) [5.9]	1.8 (2.0) [5.1]	0.4 (1.1) [2.3]	0.09 (0.4) [0.8]	0.07 (0.4) [0.7]	0.02 (0.2) [0.3]
Proportion of forced allocations	0 (0) [0]	0 (0) [0]	0 (0) [0]	0 (0) [0]	0 (0) [0]	0 (0) [0]	0.05 (0.03) [0.10]	0.03 (0.02) [0.07]	0.00 (0.01) [0.02]	0.04 (0.02) [0.08]	0.02 (0.02) [0.05]	0.00 (0.01) [0.02]
Proportion of patients sent home[1]	0.11 (0.06) [0.21]	0.05 (0.04) [0.11]	0.01 (0.02) [0.04]	0.04 (0.03) [0.09]	0.02 (0.02) [0.06]	0.00 (0.01) [0.02]	0 (0) [0]	0 (0) [0]	0 (0) [0]	0 (0) [0]	0 (0) [0]	0 (0) [0]
Drug overage	0.53 (0.04) [0.60]	0.88 (0.06) [0.98]	1.19 (0.06) [1.29]	0.53 (0.04) [0.60]	0.88 (0.06) [0.98]	1.19 (0.06) [1.29]	0.49 (0.05) [0.57]	0.84 (0.07) [0.96]	1.18 (0.07) [1.29]	0.52 (0.05) [0.60]	0.88 (0.06) [0.98]	1.19 (0.06) [1.29]
Number of patients waitlisted[2]	0 (0) [0]	0 (0) [0]	0 (0) [0]	1.1 (1.9) [4.2]	0.3 (0.8) [1.6]	0.03 (0.2) [0.4]	3.0 (4.0) [8.6]	0.9 (1.6) [3.5]	0.1 (0.5) [0.9]	2.1 (2.9) [6.9]	0.6 (1.2) [2.6]	0.1 (0.4) [0.7]
Number of patients not allocated[3]	0 (0) [0]	0 (0) [0]	0 (0) [0]	0.02 (0.20) [0.35]	0.01 (0.09) [0.15]	0.00 (0.00) [0.00]	0.09 (0.41) [0.77]	0.03 (0.25) [0.45]	0.00 (0.04) [0.07]	0.05 (0.29) [0.53]	0.01 (0.15) [0.26]	0.00 (0.01) [0.02]
Time (days) to complete recruitment	157 (26) [200]	152 (27) [196]	149 (27) [194]	152 (27) [196]	150 (27) [195]	149 (27) [193]	148 (27) [193]	148 (27) [193]	148 (27) [193]	148 (27) [193]	148 (27) [193]	148 (27) [193]

[1] An eligible patient is sent home (not randomized), if there is a lack of a given drug supply at the study site (applies to FR0a and FR0b only).

[2] In a situation when there is no drug available at the site, a patient is called to stay at home, and they are subsequently allocated to treatment once the drug supply arrives to the site.

[3] Number of patients left on the waiting list once the target recruitment is reached.

Table S5: Summary statistics - Mean (SD) [Q90] - of design operating characteristics under the "slower recruitment" scenario (with mean recruitment rate of 0.3 patients per center per week, which corresponds to a Poisson-gamma model with λ_i~Gamma(1.2,28)), for 12 different combinations of an IRT configuration (FR0a, FR0b, FR1a, FR1b) and a re-supply strategy (Low, Medium, High) , with N=16 centers and n=100 patients.

IRT configuration	FR0a			FR0b			FR1a			FR1b		
Re-supply strategy	Low	Medium	High	Low	Medium	High	Low	Medium	High	Low	Medium	High
Imbalance, |D|	0 (0) [0]	0 (0) [0]	0 (0) [0]	0 (0) [0]	0 (0) [0]	0 (0) [0]	1.6 (1.9) [4.6]	0.9 (1.6) [3.5]	0.1 (0.6) [1.1]	0.03 (0.3) [0.5]	0.02 (0.2) [0.4]	0.00 (0.1) [0.1]
Proportion of forced allocations	0 (0) [0]	0 (0) [0]	0 (0) [0]	0 (0) [0]	0 (0) [0]	0 (0) [0]	0.03 (0.03) [0.07]	0.01 (0.02) [0.04]	0.00 (0.00) [0.01]	0.02 (0.02) [0.04]	0.01 (0.01) [0.03]	0.00 (0.00) [0.01]
Proportion of patients sent home[1]	0.04 (0.03) [0.10]	0.02 (0.02) [0.05]	0.00 (0.01) [0.01]	0.02 (0.02) [0.05]	0.01 (0.01) [0.03]	0.00 (0.00) [0.01]	0 (0) [0]	0 (0) [0]	0 (0) [0]	0 (0) [0]	0 (0) [0]	0 (0) [0]
Drug overage	0.57 (0.04) [0.63]	0.91 (0.06) [1.00]	1.22 (0.06) [1.32]	0.57 (0.04) [0.63]	0.91 (0.06) [1.00]	1.22 (0.06) [1.32]	0.54 (0.05) [0.62]	0.88 (0.07) [0.99]	1.22 (0.06) [1.32]	0.57 (0.04) [0.64]	0.91 (0.06) [1.00]	1.22 (0.06) [1.31]
Number of patients waitlisted[2]	0 (0) [0]	0 (0) [0]	0 (0) [0]	0.23 (0.7) [1.3]	0.05 (0.3) [0.5]	0.00 (0.1) [0.1]	0.78 (1.4) [3.1]	0.18 (0.7) [1.3]	0.01 (0.1) [0.3]	0.45 (1.0) [2.1]	0.10 (0.4) [0.8]	0.01 (0.1) [0.2]
Number of patients not allocated[3]	0 (0) [0]	0 (0) [0]	0 (0) [0]	0.00 (0.04) [0.06]	0.00 (0.01) [0.02]	0.00 (0.01) [0.02]	0.00 (0.15) [0.26]	0.00 (0.05) [0.08]	0.00 (0.02) [0.03]	0.00 (0.09) [0.15]	0.00 (0.03) [0.05]	0.00 (0.01) [0.02]
Time (days) to complete recruitment	220 (42) [289]	217 (42) [286]	214 (43) [285]	216 (42) [286]	215 (43) [285]	214 (43) [285]	214 (43) [285]	214 (43) [285]	214 (43) [285]	214 (43) [286]	214 (43) [285]	214 (43) [285]

[1] An eligible patient is sent home (not randomized), if there is a lack of a given drug supply at the study site (applies to FR0a and FR0b only).

[2] In a situation when there is no drug available at the site, a patient is called to stay at home, and they are subsequently allocated to treatment once the drug supply arrives to the site.

[3] Number of patients left on the waiting list once the target recruitment is reached.

Table S6: Summary statistics - Mean (SD) [Q90] - of design operating characteristics under the "faster recruitment" scenario (with mean recruitment rate of 1.68 patients per center per week, which corresponds to a Poisson-gamma model with λ_i~Gamma(1.2,5)), for 12 different combinations of an IRT configuration (FR0a, FR0b, FR1a, FR1b) and a re-supply strategy (Low, Medium, High) , with N=16 centers and n=100 patients.

**IRT configuration**	**FR0a**			**FR0b**			**FR1a**			**FR1b**		
**Re-supply strategy**	**Low**	**Medium**	**High**	**Low**	**Medium**	**High**	**Low**	**Medium**	**High**	**Low**	**Medium**	**High**
Imbalance, |D|	0 (0) [0]	0 (0) [0]	0 (0) [0]	0 (0) [0]	0 (0) [0]	0 (0) [0]	2.9 (2.3) [6.6]	3.1 (2.5) [7.3]	2.2 (2.2) [5.9]	0.2 (0.7) [1.3]	0.3 (0.7) [1.5]	0.1 (0.6) [1.1]
Proportion of forced allocations	0 (0) [0]	0 (0) [0]	0 (0) [0]	0 (0) [0]	0 (0) [0]	0 (0) [0]	0.10 (0.03) [0.15]	0.08 (0.04) [0.14]	0.04 (0.03) [0.09]	0.09 (0.03) [0.15]	0.08 (0.04) [0.15]	0.04 (0.03) [0.09]
Proportion of patients sent home[1]	0.48 (0.21) [0.83]	0.25 (0.13) [0.47]	0.10 (0.09) [0.25]	0.15 (0.07) [0.27]	0.11 (0.07) [0.22]	0.05 (0.05) [0.12]	0 (0) [0]	0 (0) [0]	0 (0) [0]	0 (0) [0]	0 (0) [0]	0 (0) [0]
Drug overage	0.48 (0.05) [0.56]	0.86 (0.06) [0.96]	1.15 (0.07) [1.26]	0.46 (0.05) [0.55]	0.85 (0.07) [0.96]	1.14 (0.07) [1.25]	0.45 (0.06) [0.54]	0.82 (0.07) [0.94]	1.12 (0.08) [1.24]	0.47 (0.05) [0.56]	0.85 (0.07) [0.96]	1.14 (0.07) [1.25]
Number of patients waitlisted[2]	0 (0) [0]	0 (0) [0]	0 (0) [0]	12.9 (10.3) [30.0]	4.3 (5.4) [13.3]	1.5 (3.1) [6.5]	22.1 (13.2) [43.9]	9.8 (8.4) [23.6]	3.4 (5.1) [11.8]	20.5 (13.4) [42.5]	8.2 (8.1) [21.5]	3.0 (4.8) [10.9]
Number of patients not allocated[3]	0 (0) [0]	0 (0) [0]	0 (0) [0]	0.69 (1.77) [3.6]	0.16 (0.74) [1.4]	0.05 (0.48) [0.8]	1.4 (2.5) [5.5]	0.45 (1.3) [2.6]	0.13 (0.7) [1.3]	1.24 (2.4) [5.2]	0.32 (1.1) [2.1]	0.10 (0.6) [1.1]
Time (days) to complete recruitment	95 (17) [122]	87 (18) [116]	81 (18) [111]	84 (19) [114]	82 (19) [112]	79 (19) [110]	78 (19) [110]	77 (19) [109]	77 (19) [109]	78 (19) [110]	77 (19) [109]	77 (19) [109]

[1] An eligible patient is sent home (not randomized), if there is a lack of a given drug supply at the study site (applies to FR0a and FR0b only).

[2] In a situation when there is no drug available at the site, a patient is called to stay at home, and they are subsequently allocated to treatment once the drug supply arrives to the site.

[3] Number of patients left on the waiting list once the target recruitment is reached.
